# Nucleo-cytoplasmic compatibility in interspecies *Saccharomyces* hybrids and the destabilisation of the mitogenome by allospecific recombination

**DOI:** 10.1038/s41598-026-51924-x

**Published:** 2026-05-09

**Authors:** Zsuzsa Antunovics, Viktoria Hodorová, Jozef Nosek, Matthias Sipiczki

**Affiliations:** 1 Department of Genetics and Applied Microbiology, University of Debrecen, Debrecen, 4032 Hungary; 2 Department of Biochemistry, Faculty of Natural Sciences, Comenius University Bratislava, Bratislava, 84215 Slovakia

**Keywords:** *Saccharomyces*, Interspecies hybrid, Mitogenome, Recombination, Biological isolation, Genomics, Evolution, Genetics

## Abstract

The postzygotic reproductive isolation of species in the *Saccharomyces* genus is maintained by failure of the homeologous chromosomes to pair in meiosis and by repression of the mating programme by *MATa/MATalpha* heterozygosity, but nucleo-mitochondrial incompatibilities causing respiration deficiency were also proposed to be involved. In this study, we investigated the compatibility of parental and recombinant mitogenomes with alloploid nuclear genomes in synthetic *S. cerevisiae* x *S. uvarum* hybrids. All hybrids were homoplasmic but their mitogenomes showed high structural diversity due to frequent allospecific recombination. 34 recombination sites were identified, all in coding regions. From the location of the sites, it could be inferred that both intron-homing and crossing-over were involved. The recombinant mitogenomes had diverse combinations of parental and chimeric genes. The respiration proficiency of the hybrids indicates compatibility between their (parental or recombinant) mitogenomes and alloploid nuclear genomes. However, the recombinant mitogenomes were unstable, resulting in respiration-deficient segregants in the hybrid cultures. Since lack of respiration impairs sporulation efficiency and also competitiveness in mixed populations with respiration-proficient (e.g. parental) cells, destabilisation of the mitogenome by allospecific recombination may (indirectly) contribute to the biological isolation of *Saccharomyces* species.

**Supplementary Information:**

The online version contains supplementary material available at 10.1038/s41598-026-51924-x.

## Introduction

Reproductive isolation maintains the integrity of species by preventing (or at least reducing) gene flow between them. The isolation mechanism can be prezygotic, which prevents the species from producing offspring, or postzygotic, which allows hybrid formation but the hybrids are sterile, incapable of producing functional gametes (sterility barrier)^1^. Hybrid sterility can have complex causes in certain groups of organisms, but is mainly due to differences between the homeologous chromosomes of the hybridising partners, which prevent their efficient pairing in the prophase of meiosis I (e.g.^2–7^). The failure of chromosome pairing results in the collapse of the meiotic division machinery or at least in the production of gametes with aberrant, non-functional sets of chromosomes. In many groups of plant species and in certain animal genera, the postzygotic sterility barrier can be circumvented by whole-genome duplication (WGD), which occurs spontaneously in allodiploid hybrids. WGD diploidises the parental subgenomes, within which each chromosome will have a homologous partner to pair with during meiosis. This process referred to as the autodiploidisation of allotetraploid meiosis^8^ results in the formation of fertile allodiploid gametes that can mate with other allodiploid gametes to produce fertile allotetraploid progeny (restoration of fertility by genome duplication). This phenomenon can have evolutionary consequences because new species and evolutionary lineages can evolve from fertile allotetraploid hybrids (alloploid hybrid speciation) (e.g.^9–12^). Many plant species are thought to have evolved from interspecies hybrids (e.g.^9^).

The species of the budding yeast genus *Saccharomyces* are also isolated postzygotically but the mechanism of the barrier is more complex. Like certain closely related plant and animal species, the *Saccharomyces* species can form viable but sterile allodiploid hybrids. The sterility is manifested in their inability to form viable ascospores, the yeast equivalents of plant and animal gametes. Meiosis is lethal to these cells, but they can be rescued by forced “return-to-mitotic-growth“^13^. However, in contrast to sterile plant and animal hybrids, the *Saccharomyces* hybrids do not become fertile when their genomes are duplicated. Genome duplication does not restore fertility. It only restores spore (gamete) viability (e.g.^14^) because the allodiploid spores cannot mate (fertilise)^15^. These spores are sterile because they receive different *MAT* loci from the parental subgenomes (*MATa* from one subgenome and *MATalpha* from the other subgenome)^16^. *MATa/MATalpha* heterozygosity represses the mating-specific genes (for a review of the genetic determination of sexual processes in *Saccharomyces*, see^17^). Consequently, it is extremely rare for such a heterozygote to conjugate with another cell, and such an event is presumably due to accidental inactivation of one or other *MAT* locus^18^. The *Saccharomyces* species are thus reproductively isolated by two sterility barriers, one of which acts in allodiploid hybrids and the other upon genome duplication. The latter does not exist in plants and animals because the sexual processes of plants and animals are determined in different ways. Sex determination based on an active locus (*MAT*) and two silent loci (*HMRa* and *HMLalpha*) as well as the alternative activation of the mating programme and the sporulation (gametogenesis) programme by the *MAT* locus^17^, is specific to *Saccharomyces* and related genera.

In contrast to the presence of both parental subgenomes in the hybrid *Saccharomyces* nucleus, the hybrid cytoplasm typically has only one type of mitochondrial genome. In most cases one of the parental mitotypes predominates, but recombinant mitotypes were also detected in synthetic hybrids (e.g.^19–29^). In a recent study, we identified recombinant mitochondrial DNA (mtDNA) in 34% of *S. cerevisiae* x *S. uvarum* (cevarum) hybrids^30^. These hybrids appeared to be homoplasmic but their mtDNAs were highly diverse in the RFLP analysis. Given this diversity of mitogenomes in hybrids, the question arises as to whether any parental or recombined mitotype can affect the fertility of the hybrid cells. In other words, can nucleo-mitochondrial interactions contribute to hybrid sterility? This possibility has been addressed by several studies. The results of those studies revealed that disturbances in the coordinated activity of certain nuclear and mitochondrial genes, which lead to the cessation of cellular respiration, can simultaneously prevent the formation of viable ascospores. Respiration-deficient (*petite*) cells cannot sporulate (e.g.^31^) because respiration is necessary for the activation of *IME1*, a gene encoding a regulator of meiosis and sporulation^32^. Numerous incompatible combinations of nuclear and mitochondrial genes of different *Saccharomyces* species have been described, but remarkably, not in “true” (allodiploid) hybrids but in their alloaneuploid descendants with incomplete sets of parental chromosomes or haploid segregants with mixed (chimeric) sets of parental chromosomes (the complete nuclear genome structure was not always clearly described). For example, in an alloaneuploid segregant of a cevarum hybrid with the *S. cerevisiae* mitotype, the lack of the *S. cerevisiae AEP2* gene in the nucleus caused respiration deficiency because its *S. uvarum* counterpart was incompatible with the *oli1/atp9* gene of the *S. cerevisiae* mitochondrion^21^. The protein encoded by the *S. uvarum* nuclear gene *AEP2* cannot regulate the translation of the *S. cerevisiae* mitochondrial atp9 mRNA. Incompatibility was also revealed between the *S. uvarum* nuclear gene *CCM1* and the *S. cerevisiae* mitochondrion^33^. Cevarum hybrids with *S. uvarum* mitotypes but lacking the *S. uvarum* nuclear genes *MRS1* or *AIM22* were respiration-deficient because their *S. cerevisiae* orthologues could not substitute them^34^. The *S. cerevisiae* Mrs1 protein cannot correctly splice the *S. uvarum* cox1 transcript. However, these incompatibilities are unlikely to contribute to hybrid sterility, as all mitochondrial genes have compatible (conspecific) partners in one or the other subgenome of the allodiploid nuclear genome. The mitogenomes of *Saccharomyces* interspecies hybrids and their segregants were also investigated from other aspects. For example, their influence on phenotypic traits (e.g. cold and heat tolerance, growth rate, respiration power and QTL landscape) was investigated in recent studies (e.g.^14,24,29,35,36^).

The aim of this study is to investigate the co-operation of parental and recombinant mitogenomes with the alloeuploid nuclear genome in synthetic hybrids of *S. cerevisiae* and *S. uvarum* with regard to the biological isolation of these distantly related species (e.g.^37^) of the genus. All synthetic hybrids were homoplasmic, either for parental or for recombinant mitogenomes. The sequence analysis of the recombinant mitogenomes identified 34 sites at which the (allospecific) parental mtDNAs recombined. All sites were located in coding regions or in close vicinity to *trn* genes. Their locations indicate that both intron homing and crossing over were involved in recombination. All combinations of the parental and chimeric mitochondrial genes were compatible with the hybrid nuclei, suggesting that nucleo-cytoplasmic incompatibility does not play a significant role in the biological isolation of these species. However, the destabilising effect of recombination on the mitogenomes may contribute to it. The recombinant mitogenomes were prone to segregation leading to loss of respiration competence, which is a disadvantage in competition with the parental strains.

## Results

### Isolation and phenotypic characterisation of alloploid hybrids

One hundred prototrophic colonies as putative interspecies cevarum hybrids were isolated from the intersections of prints of the parental cultures on the selective medium (on this medium the auxotrophic parental strains did not grow). To distinguish them from hybrids produced in our previous studies (labelled as A, Cu and S), their identification codes start with “B” in Table 1. All hybrids could utilise glycerol as a carbon source at 25 °C and inherited the ability of the *S. cerevisiae* parent to grow at 37 °C and the ability of the *S. uvarum* parent to utilise melibiose. Since glycerol is a non-fermentable carbon source, the ability of the hybrids to utilise it indicates that their mitochondria were functional. On the sporulation medium all hybrids formed spores. All had “cevarum-type” alloploid karyotypes with complete sets of parental chromosomal bands (Supplementary Fig. 1S). Diversity was observed only between the bands of Chr. XII of *S. cerevisiae* and Chr. 15 of *S. uvarum* (the *S. uvarum* chromosomes are numbered according to^38^), where certain hybrids had a faint band. As this band had no counterpart in the parental karyotypes, it could be an artefact generated during the preparation of the samples for karyotyping or the extension of the rDNA array upon hybridisation^39^.

**Table 1:** List of strains.

Strain		Features	Source
Identification number	Species		
10–170	*S. cerevisiae*	*MATa leu2*, heat tolerant, mel^-^	ATCC 204,891, (YGSC X4005-11A)
10–522	*S. uvarum*	*HO ura3*, heat sensitive, mel^+^	Antunovics et al., 2005
A4, A27, A33, A35, A36, A37, A38	“cevarum” hybrids 10–170 × 10–522	Prototrophic	Szabo et al., 2020
Cu4.1, Cu4.8			
S2, S6, S15, S24			
B1 to B100	“cevarum” hybrids 10–170 × 10–522	Prototrophic	This study

### Parental and non-parental mitochondrial RFLP patterns in hybrids

The *Mbo*I digestion of the mtDNA of the hybrids produced in this study revealed both parental and non-parental restriction patterns. Seventy-nine hybrids had patterns indistinguishable from that of the *S. cerevisiae* parent. Only two hybrids showed patterns identical to that of the *S. uvarum* parent. The pattern identity suggested homoplasmy. Nineteen hybrids differed from both parents in banding (Supplementary Fig. 2S). Their patterns consisted of fragments corresponding in size to certain fragments of the parental patterns and non-parental fragments. Therefore these mitochondrial genomes were considered recombinant. As none of them contained a complete parental set of bands, these hybrids were not heteroplasmic for parental and recombinant mitogenomes. Their patterns also differed from each other (Supplementary Fig. 3S), indicating that the parental mitochondrial genomes recombined in multiple ways. Interestingly, 5 of these hybrids and 9 hybrids from our previous study^30^, which exhibited non-parental RFLP patterns, did not grow on glycerol at 37 °C, indicating temperature-sensitive respiratory deficiency. The hybrids which were sequenced (see later) are listed in Supplementary Table 1S.

### Structural diversity of non-parental (recombinant) mitochondrial genomes

**Figure 1:**
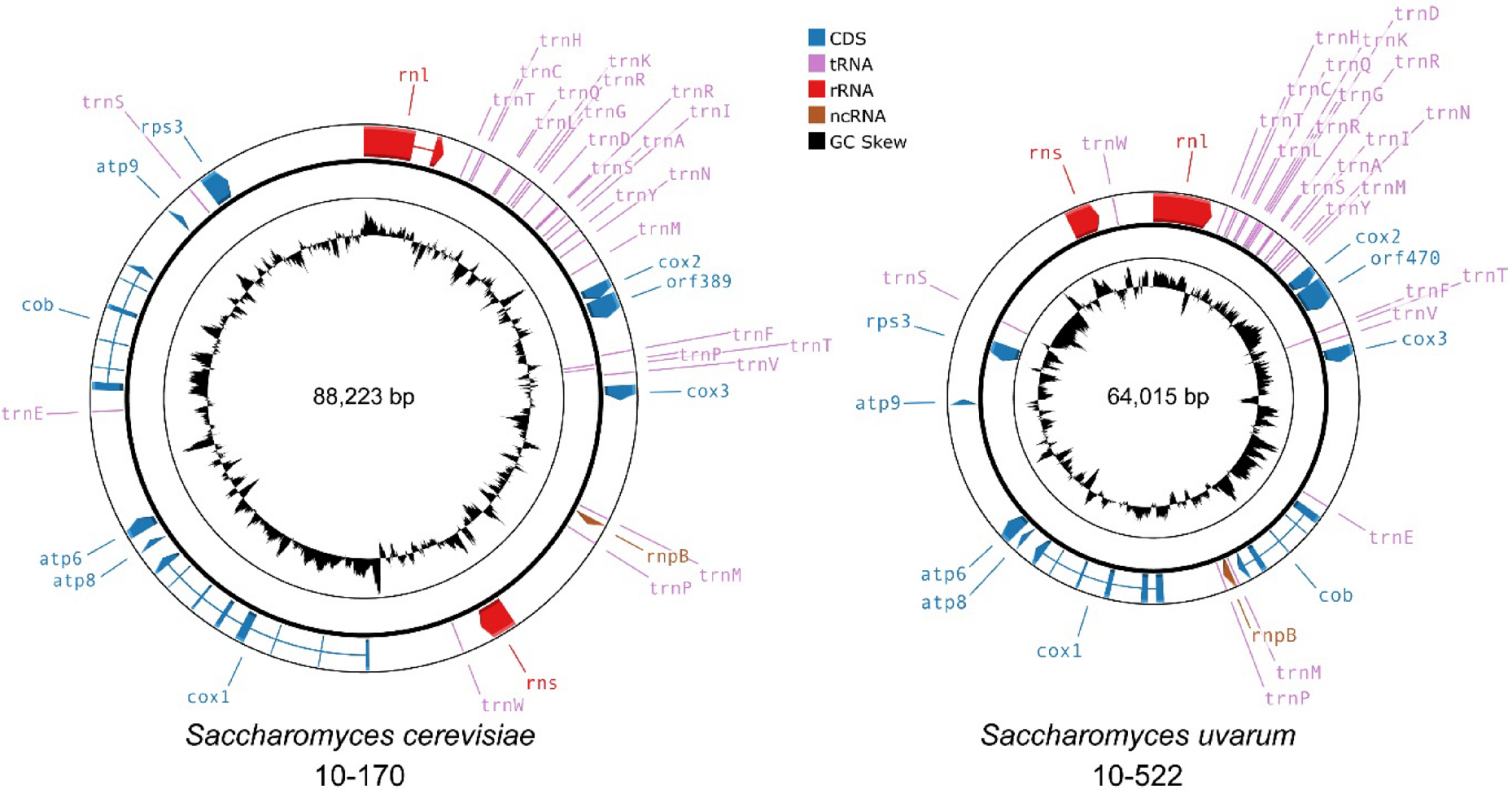
Circular maps of the mitochondrial genomes of the hybridisation partners.

**Figure 2:**
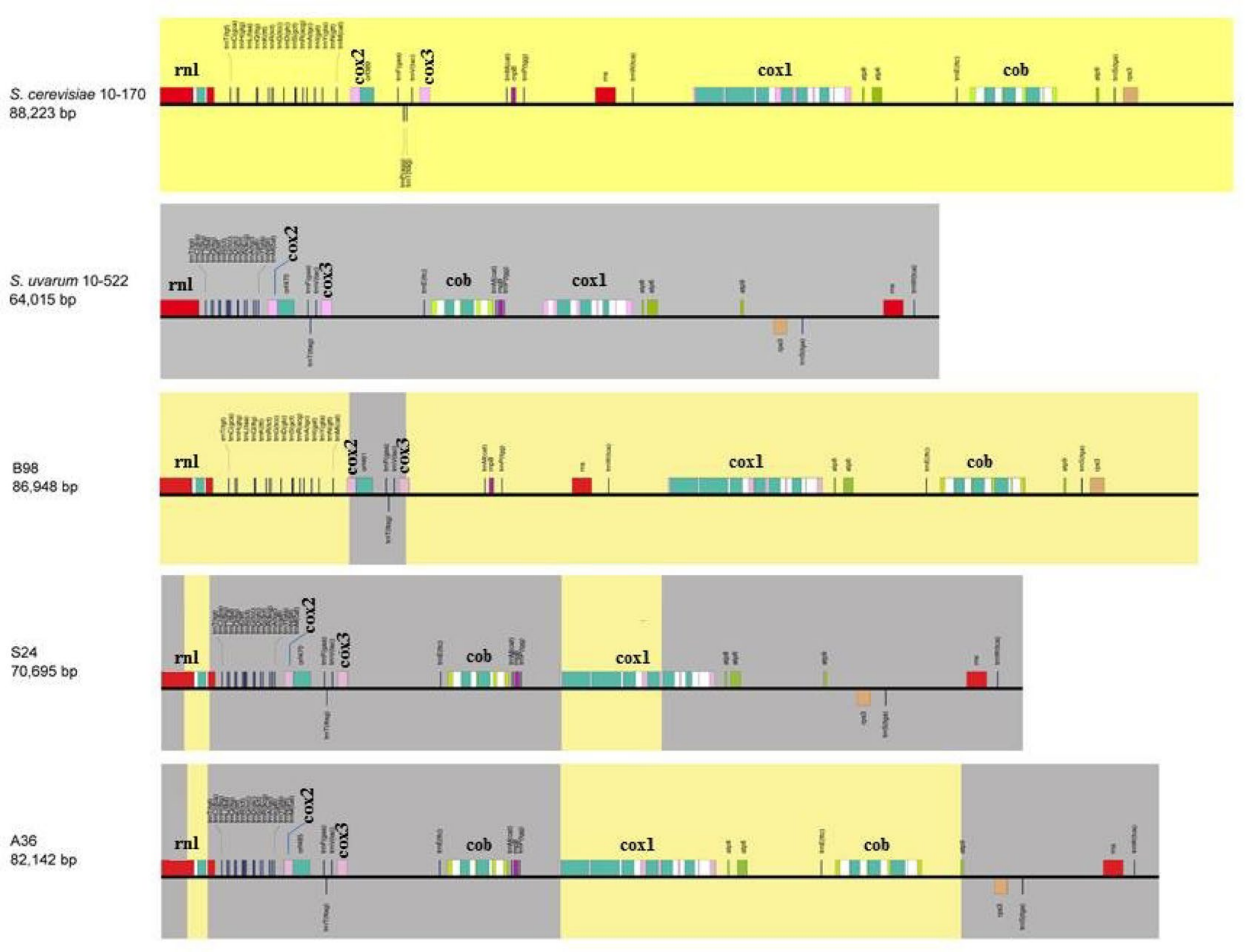
Examples of linear maps of recombinant (chimeric) mitogenomes. B98 is a recombinant genome in which the *S. cerevisiae* mtDNA predominates. S24 is a recombinant genome in which the *S. uvarum* mtDNA predominates. The A36 genome contains a duplication. The number of recombination sites is two in B98 and four in S24 and A36. Yellow: *S. cerevisiae* sequence. Grey: *S. uvarum* sequence. All linear maps are shown in Supplementary Fig. 6S.

To investigate the mitogenomes in more detail, we determined the complete mtDNA sequences of both parental strains and 28 hybrids (from this work and our previous study) exhibiting recombinant RFLP patterns. The homoplasmy assay by aligning the sequencing reads to the mitochondrial genome sequences confirmed the results obtained by RFLP that the recombinant hybrids were also homoplasmic (Supplementary Fig. 4S). In the histogramme of the hybrid A27, the coverage was heterogeneous. This can be due either to heteroplasmy or to the presence of cells with segregant mitogenomes in the culture from which DNA was extracted for sequencing. The latter possibility is more likely due to the instability of the recombinant mitogenomes (see below). The mitochondrial genomes of the parental strains had the size, GC content and structural characteristic of their species^40,41^ with pairwise sequence identity to the reference sequences of about 93% in both cases. The *S. uvarum* 10-522 mitogenome (64 015 bp) was smaller and more compact than the *S. cerevisiae* 10-170 mitogenome (88 223 bp) mostly because of shorter intergenic regions and fewer introns (Fig. 1). The recombinant mitochondrial genomes were highly diverse in size (Supplementary Fig. 5S) and composition as illustrated by linear maps shown in Fig. 2 and Supplementary Fig. 6S, in which the parental segments are marked with different colours. Each strain had a unique combination of parental segments. Although the ratio of the parental mitogenomes in the individual hybrid genomes was highly diverse (Supplementary Fig. 5S), the parental contribution to the chimeric genomes was almost identical when all recombinants were considered. The average OrthoANIu value was 94.97 compared with the *S. cerevisiae* parent and 93.31 compared with the *S. uvarum* parent (*P* = 0.4653). The averages of the gANI values were also only slightly different (*P* = 0.1997). Both ANI and OrthoANI are computational metrics used to measure nucleotide-level similarity between two genome sequences. Both calculate mean sequence identity of fragments after fragmentation of both genome sequences. ANI calculates it from all compared fragment pairs, while OrthoANI considers only orthologous fragment pairs (reciprocal best-hit pairs). A clear trend can be seen. The smaller the genome is, the greater the proportion of the *S. uvarum* mtDNA, and conversely, the larger the genome is, the greater the proportion of the *S. cerevisiae* mtDNA. This observation suggests that the recombinant mitochondrial genomes remains functional even when large parts of the intergenic regions of one or the other parent are missing.

### Recombination took place in multiple sites

**Figure 3:**
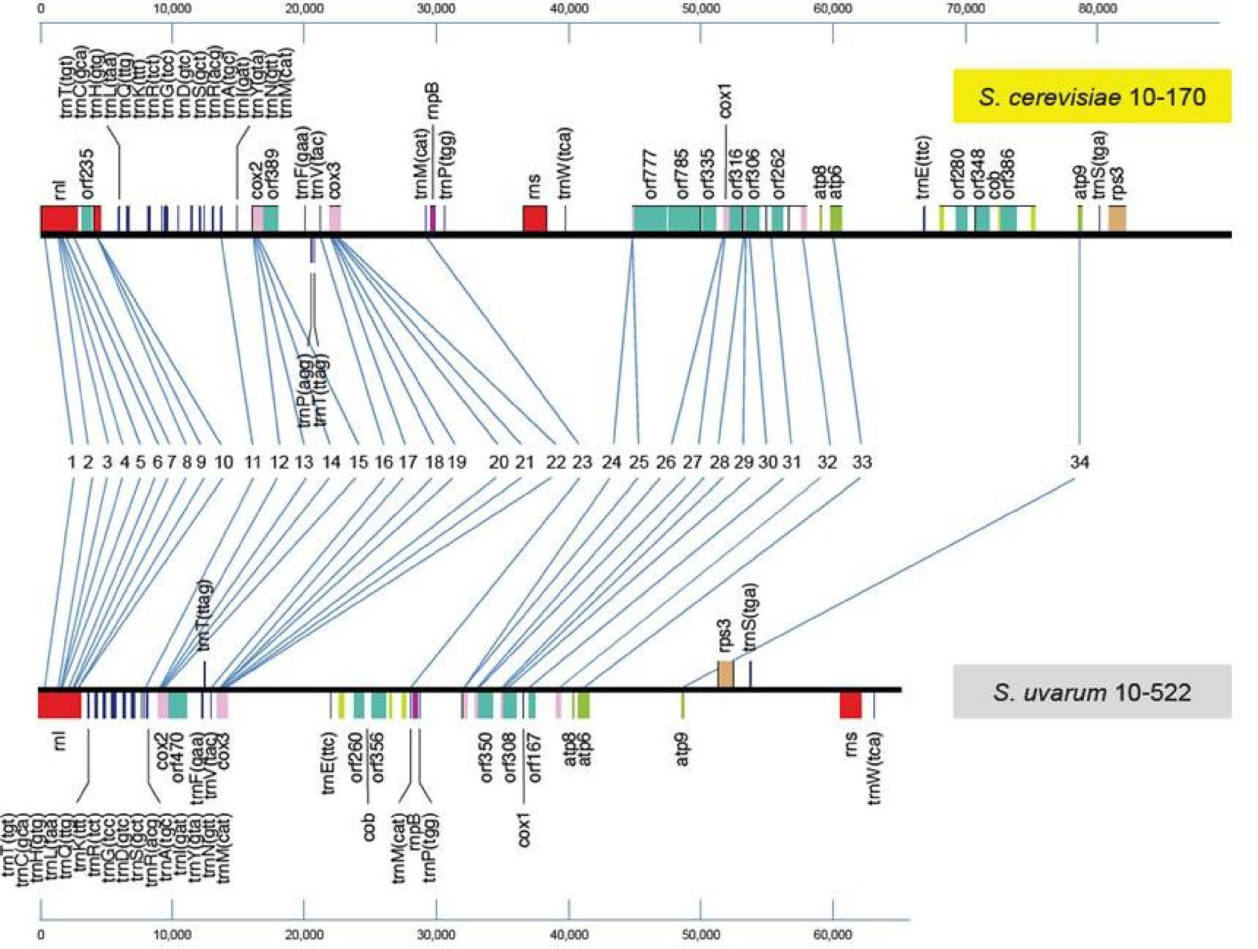
Locations of the recombination sites in the parental mitogenomes. The converging lines mark sites that are close to each other.

Comparison of the recombinant and parental mitogenomes using Mauve alignments identified 34 sites, where recombination between the parental mtDNAs had taken place (Fig. 3). At these sites the parental genomes shared identical sequences varying in size from 2 to 247 nucleotides (Supplementary Table 2S). The recombination sites were unevenly distributed and grouped into 9 segments. Ten were located within the *rnl* gene. Three sites overlapped with the tRNA genes *trnN*, *trnV*, *trnY* and *trnM*. The remaining sites were located in protein-encoding genes (*cox1*, *cox2*, *cox3*, *atp6* and *atp9*). Two sites (30 and 31) were found in introns. No recombination sites were found in genes *cob*, *rps3, rnpB* and *rns* (Fig. 3). None of the sites overlap with GC-rich clusters and none of them coincide with segments similar to the mitochondrial *ori* sequences of *S. cerevisiae* S288c. However, it has to be mentioned that the latter segments of the *S. cerevisiae* strain used in this study show only 75–85% sequence identity with the S288c *ori* sequences. Remarkably, no recombination sites were found in the regions separating the protein-encoding genes in the parental mitogenomes. The Blast similarity search with these sequences of one parent in the mitogenome of the other parent revealed no significant similarity or only very low degree of sequence identity (Table 2).

**Table 2:** Similarity of regions separating protein-encoding genes in the parental mitogenomes.

Segment	Length (number of nucleotides) in		Blast comparison (region against genome)	
	*S. cerevisiae* 11–170	*S. uvarum* 11–522	Coverage (%)	Identity (%)
Colinear				
*Cox2-Cox3*	4921	3646	89	67.62
*Atp8-Atp6*	666	330	No significant similarity	
*Atp9-Rps3*	1997	2524	No significant similarity	
*Cox1-Atp8*	965	797	No significant similarity	
Non-colinear				
*Cob-Cox1*	–	4192	26	75.87
*Cox3-Cob*	–	8230	14	73.68
*Atp6-Atp9*	–	6831	84	73.93
*Cox1-Atp8*	965	–	No significant similarity	
*Atp6-Cob*	7275	–	7	73.68
*Cob-Atp9*	3220	–	No significant similarity	

### Coincidence of recombination sites with sites of intron homing

**Figure 4:**
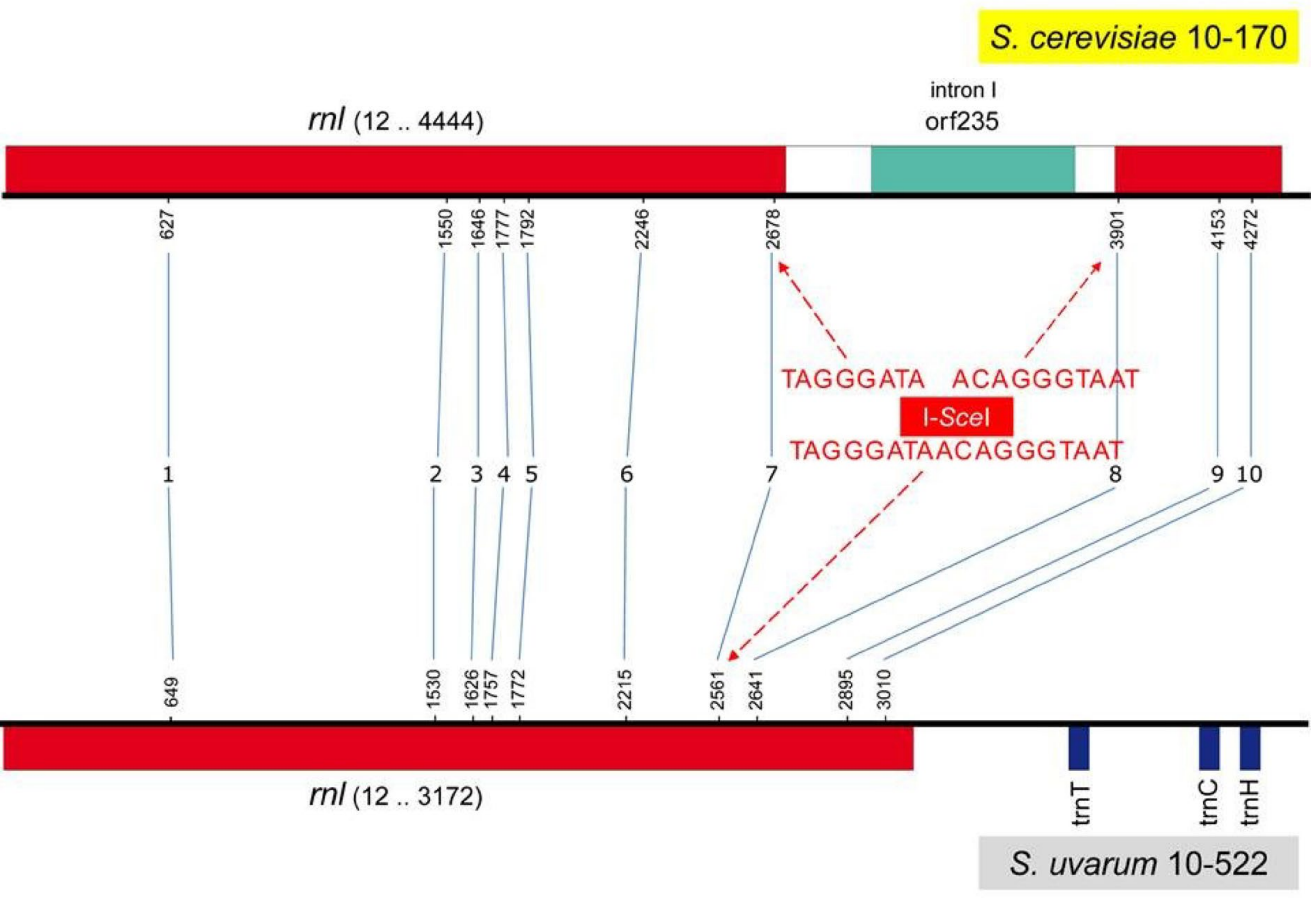
Locations of recombination sites in the *rn1* segments of the parental strains. The sequence of the cleavage site of I-*Sce*I is shown in red.

Intron homing is a process in which the intron moves from an intron-containing allele to an intron-less allele in a gene conversion event. The process is initiated by generating a double-strand break in the recipient allele by the homing endonuclease encoded by the donor intron. Homing is accompanied by the co-conversion of flanking sequences that can be several kbp long (for a review, see^42^). A search in the parental genomes identified several orfs coding for homing endonucleases in the *S. cerevisiae* genome. *orf235* located in the intron of *rn1* codes for I-*Sce*I. The sequence of its cleavage site is disrupted by the intron. The mitogenome of the *S. uvarum* parent has an unsplit equivalent of this sequence at a homologous position (Fig. 4). Recombination sites 7 and 8 are located in close vicinity to this sequence in both parental genomes.

**Figure 5:**
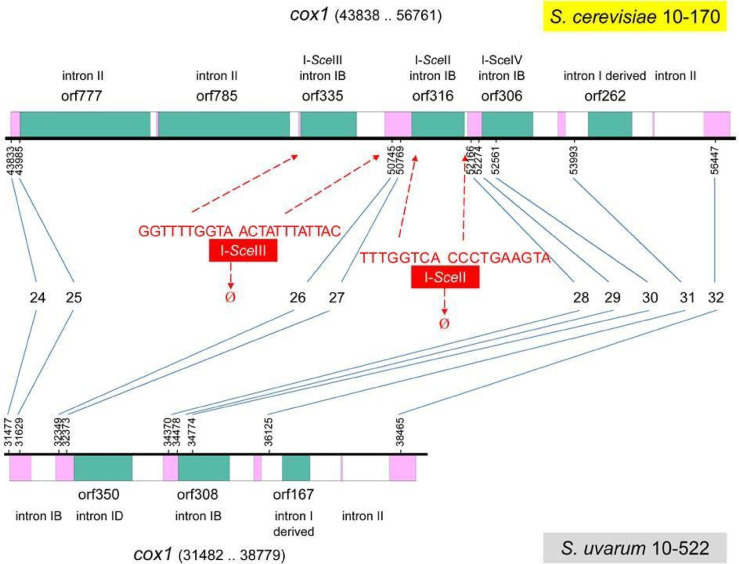
Locations of recombination sites in *cox1* of the parental strains. The sequences of the cleavage sites of I-*Sce*III and I-*Sce*II are shown in red. The classification of introns and intronic ORFs is based primarily on the MFAnnot predictions^69^. The ORF numbering reflects the number of codons in the corresponding reading frames. I-SceII, I-Sce-III, and I-SceIV are intron-encoded endonucleases.

The introns of *cox1* harbour *orf335*, *orf316* and *orf305*. *orf335* and *orf316* encode I-*Sce*III and I-*Sce*II, respectively. Their split cleavage sequences flank the housing introns. The distances separating them from the nearest recombination sites (located in the adjacent exons) were 186 and 117 nucleotides long indicating that the recombination events at these sites were not initiated by I-*Sce*III or I-*Sce*II. Their involvement is also unlikely because we could not find their recognition sequences in the *cox1* gene of the *S. uvarum* mitogenome (Fig. 5). *orf305* is located in intron 5 of *cox1* of the reference strain S288c and many other *S. cerevisiae* strains. It encodes the I-*Sce*IV homing endonuclease which is thought to be involved in the integration of copies of the housing intron into the sequence 5′ AAAATCTTTTCTTGATTAGCCCTAATCTACGGT 3′ of intronless positions^43^. Neither *cox1* gene of the parental mitochondrial genomes of the cevarum hybrids examined in this study has this or a similar sequence. Intron 3 of *cox1* of *S. uvarum* 10–522 is highly similar (90.02% identity) to intron 5 of *S. cerevisiae* 10–170 and contains an orf (*orf* 308) that codes for a putative homologue of I-*Sce*IV (91.43% sequence identity). Owing to the presence of this orf and the lack of a proper integration site in intron 3 of 10–522, recombination initiation by an I-*Sce*IV-driven homing event is unlikely. Thus, the recombination at site 30 (Fig. 5) can be attributed to crossover between identical stretches of *orf306* (*S. cerevisiae*) and *orf308* (*S. uvarum*) (Supplementary Table 2S) rather than to intron homing.

### Diverse combinations of parental and chimeric genes in the recombinant genomes

All but two recombinant mitogenomes had only one copy of each gene (Supplementary Fig. 7S), either from one or the other parental genome but certain genes were chimeras of the parental genes. The exceptions were the recombinant genomes A36 and B25, which possessed two *cob* genes, one from each parent. The co-existence of both parental genes can be attributed to recombination (crossing-over) between segments of the parental genomes in which the genes are in reverse order (see Figs. 1, 2). The proteins encoded by the chimeric *cox1*, *cox2* and *atp6* genes and the majority of the chimeric Cox3 proteins are more similar to the *S. uvarum* proteins, whereas the amino acid sequence encoded by the chimeric *atp9* gene is identical to that of the *S. cerevisiae* parent (Supplementary Fig. 8S).

### Hybrids with recombinant mitochondrial genomes form respiratory-deficient (petite) segregants

In an alloploid cell, the mitochondrion must cooperate with two different sets of nuclear genes. To test whether the presence of two different nuclear genomes can adversely affect the stability of the mitochondrial genome, cells of the hybrids were plated out on a medium containing glucose, a fermentable carbon source. All hybrids that had parental mitotypes formed colonies that were uniform in size. In contrast, the hybrids, whose mitochondrial genomes were recombined, formed mixed populations of larger (*grande*) and smaller (*petite*) colonies (Supplementary Fig. 9S). When representatives of the *grande* and *petite* colonies were tested for growth at 25 °C and 37 °C and for the ability to utilise glycerol as a non-fermentable carbon source, five phenotypes were distinguished: (a) *grande* (growth on glycerol at both temperatures), (b) temperature-sensitive *grande* (ts *grande*: no growth at 37 °C but growth on glycerol at 25 °C), (c) *petite* (no growth on glycerol), (d) temperature-sensitive *petite* (ts *petite*: growth on glycerol at 25 °C but not at 37 °C) and (e) *petite* with temperature sensitive growth (no growth at 37 °C and on glycerol at 25 °C) (Supplementary Table 1S). Certain colonies isolated as small turned out to be *grande* and a few colonies isolated as large proved to be *petite* when tested for glycerol utilisation. These isolates must have been wrongly selected. No reversion to respiratory proficiency (*grande* phenotype) was observed in *petite* segregants.

**Figure 6:**
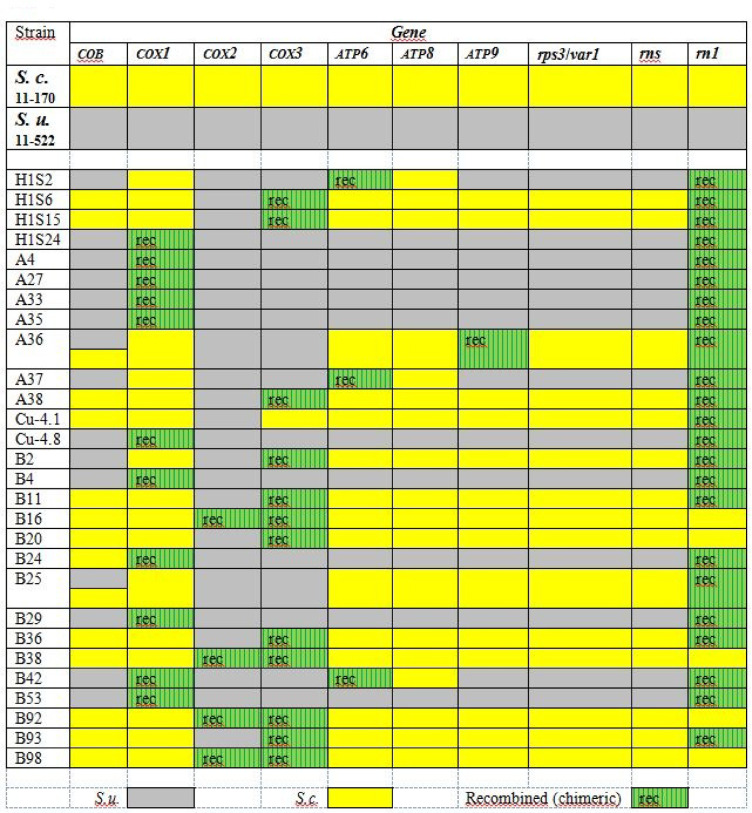
Combination of parental and chimeric genes in recombinant mitotypes.

The phenotypes “temperature-sensitive *grande*” and “*petite* with temperature-sensitive growth” could be attributed to the loss of heterozygosity (LOH) of the nuclear genes that are responsible for the difference between the parental strains in temperature tolerance^29,35,36^. Since chromosome loss can cause LOH, and because the *Saccharomyces* alloploid hybrids are prone to chromosome loss^44^, we compared the karyotypes of these segregants with the karyotypes of the hybrids from which they segregated. All had identical banding patterns that did not differ from the karyotypes of the hybrids. However, we cannot exclude changes in the segments of the karyotypes in which the chromosomal bands could not be clearly separated (Fig. 6).

### Diverse mitochondrial RFLP patterns in respiratory-deficient segregants

Two segregants isolated as small colonies (*petites*) were selected from each hybrid created in this study for RFLP analysis of their mtDNA (Supplementary Fig. 10S). Some colonies turned out to be respiration proficient as they grew on glycerol (Supplementary Table 1S). Their patterns were indistinguishable from the patterns of the hybrids. The only exception was B93/1. However, the patterns of the majority of the *petites* differed from those of the hybrids from which they segregated, and occasionally, also from each other (in hybrids B24, B33, B42, B93 and B98). In the latter hybrids intragenomic recombination leading to loss or inactivation of genes can be assumed. In contrast, certain *petite* segregants of five hybrids (B20, B25, B29, B53 and B92) showed the same patterns as their *grande* hybrid progenitors. This finding indicates that even minor changes can disrupt essential mitochondrial functions.

## Discussion

Hybridisation of a *S. cerevisiae* strain with a *S. uvarum* strain in this study and in our previous projects^30^ resulted in alloploid cevarum hybrids carrying parental or recombinant mitogenomes. Numerous previous studies of intraspecific *S. cerevisiae* hybrids have demonstrated that the zygotes are usually heteroplasmic but after ten to twenty generations, the entire hybrid culture becomes homoplasmic (e.g.^45^) It is plausible that a similar postzygotic process had taken place in the interspecies hybrids produced in this study, either during colony formation on the selective medium or later, during the recovery of the hybrids from the frozen stocks. As expected, all hybrids proved to be homoplasmic. Most of them had RFLP patterns identical to one or the other parental pattern. The rest showed different patterns, but sequencing revealed a single mitogenome in all cases. Alignments of the sequence reads with mitochondrial genome sequences in the homoplasmy tests confirmed homoplasmy in both parental strains and their hybrids that had recombinant mitogenomes.

The overwhelming majority of hybrids generated in this study received mitochondrial genomes from the *S. cerevisiae* parent. In our previous hybridisation experiments using the same parental strains and hybridisation method, the *S. cerevisiae* mtDNA was also preferentially transmitted into the hybrids (e.g.^20,30^). The bias in mitochondrial genome transmission towards one parent may be due to the formation of more efficient OXPHOS complexes and mitochondrial ribosomes when all mitochondrial proteins are produced by genes of the same parental mitogenome. As the OXPHOS complexes and mitochondrial ribosomes are products of both the nuclear and mitochondrial genes, we hypothesise that the hybrid cells in which all mitochondrial proteins are from one parental (e.g. *S. cerevisiae*) genome may have an advantage during homoplasmoidisation. However, the predominance of one of the parental mitochondrial genomes in the hybrids of a species combination is not a general rule. Other studies found that the choice of the transmitted parental mitochondrial genome may depend on environmental conditions at the time of hybrid formation^46^.

 Nineteen per cent of the hybrids produced in this study had recombinant mtDNAs. This finding is in line with our previous study reporting 34% of hybrids with recombinant mitotypes^30^. This recombination rate seems to be high when compared to those reported by other authors. Hewitt et al.^46^ detected recombined mtDNA only in one of a group of 72 *S. cerevisiae* x *S. uvarum* hybrids. Leducq et al.^47^ found that mitochondrial recombination in interspecies crosses was null or rare. Nevertheless, the high recombination frequency detected in this study demonstrates that allospecific parental mitochondria can fuse during zygote formation or shortly afterwards, in the course of “postzygotic” propagation of the hybrid cells, to allow physical interaction between the parental mtDNA molecules. Obviously, the mitochondrial fusion machineries of *S. cerevisiae* and *S. uvarum* (including the outer membrane proteins Fzo1, and Ugo1 and the inner membrane protein Mgm1, reviewed in^48^) are not incompatible.

The comparative analysis of the mitogenome sequences revealed that the *S. cerevisiae* and *S. uvarum* mitogenomes recombined within exons of genes (only two recombination sites were located in introns) or in close proximity to *trn* genes. No recombination was detected between intergenic segments, which can be attributed to the low similarity of the intergenic regions. This is an important difference from the recombination between conspecific mitogenomes which preferentially recombine in intergenic regions^49^. The locations of recombination sites also differ from those in the hybrids of *S. cerevisiae* with the phylogenetically much closer *S. paradoxus,* in which recombination mostly occurred near protein-coding exons^50^. The long *cox1* gene was home to recombination events in the hybridisation of *S. cerevisiae* with conspecific strains, with *S. paradoxus* strains and also with an *S. uvarum* strain (in this study). In the hybrids of *S. cerevisiae* with *S. paradoxus*, Hénault et al.^50^ detected recombination in the first exon, the sixth intron and at the last intron–exon junction. The last exon was also involved in the hybrids analysed by Bágeľová Poláková et al.^51^. The *cox1*-based recombination sites had different locations in our cevarum hybrids, probably due to the greater difference between the structures of the *cox1* genes of *S. cerevisiae* and *S. uvarum*. Another difference is that none of the recombination sites in the cevarum hybrids were located in GC-clusters, whereas these clusters significantly contribute to the recombination of *S. cerevisiae* mitogenomes with conspecific mitogenomes ^52–54^ or with mtDNAs of the closely related *S. paradoxus*^55^.

Mitochondrial recombination in *S. cerevisiae* has been extensively investigated for decades, yet the underlying molecular mechanisms are not fully understood. Several processes can be implicated such as recombination during replication initiation, interactions between replication intermediates (concatemers and circular monomers), gene conversion associated with intron-homing, or homologous recombination (e.g. crossing-over) between similar segments of the interacting genomes (e.g.^56,57^). In principle, any (or several) of these processes can be involved in the formation of the recombinant mitogenomes of the interspecies hybrids of this study. Recombination site 8 is located next to the recognition sequence of the homing omega endonuclease I-*Sce*I. As this sequence is also present in the intronless *S. uvarum rn1*, a homing process can be started here by cleaving the *S. uvarum* mtDNA by I-*Sce*I, and the intron homing further proceeds by recombinational repair. As the *rn1* sequences of the parental strains differ, the strand migration proceeds far away from the intron and scans the interacting mtDNAs for a region with reasonably long identical sequences to resolve the recombination intermediate. Original studies on intron homing in the *rn1* gene of *S. cerevisiae* demonstrated that recombination sites could be far from the intron insertion sites, as the single-nucleotide mutations in the *rnl* gene that confer antibiotic resistance are commonly transferred together with the intron^58^. In contrast, the recombination events in *cox1* are unlikely to be related to homing because none of the I-*Sce*II, I-*Sce*III and I-*Sce*IV endonucleases encoded by orfs of introns of this gene in the parental *S. cerevisiae* mitogenome have cleavage/integration sites in the *S. uvarum* mitogenome. Six recombination sites are located in the intronless *cox3* gene. In the *S. cerevisiae* parental genome this gene is adjacent to the stand-alone endonuclease (SAE) gene *orf106*. The product of this orf could play a role in the initiation of recombination in *cox3* because SAEs were found to increase the recombination frequency and diversity in adjacent intron-lacking genes in *S. cerevisiae*^57^. However, no copy of *orf106* is transferred into the recombinant mtDNA. Thus, the recombination events at these sites are unlikely to be initiated by this stand-alone endonuclease. The heterodimer endonuclease Endo.*Sce*I formed by the products of the nuclear gene *ENS1* and the mitochondrial gene *ENS2*^59^ could also be involved in the initiation of recombination events in certain locations. As it cleaves mtDNAs every 2–3 kb, its activity could significantly increase the number of recombination sites. However, neither the *S. cerevisiae* parent nor the *S. uvarum* parent of the hybrids analysed in this study has *ENS2*. But they have orfs (*orf389* in *S. cerevisiae* and *orf470* in *S. uvarum*) whose products show sequence similarity to homing endonucleases of the LAGLIDADG family and thus could potentially play a similar role as Ens2.

Remarkably, no recombination sites were identified in *cob*, *rns* and *rps3.* The lack of recombination in these genes can be attributed to incomplete synteny in the corresponding regions of the parental mitogenomes. Recombination between inverted sequences or their flanking segments results either in deletion (causing mitochondrial dysfunction) or in duplication. However, none of the hybrids lacked this region, and duplications were found in only two recombined genomes. It is unlikely that the duplications were created by homing, rather they were formed by crossing-over between the flanking segments. Duplications have also been observed in other species combinations. For example, Bágeľová Poláková et al.^51^ found that incomplete synteny of the mitogenomes of *S. cerevisiae* and *S paradoxus* caused a large duplication in the mitogenome of their hybrid, which covered *cox3*, *rns* and numerous *trn* genes (but not *cob*).

 The recombinant mitogenomes had virtually complete sets of genes but with diverse representations of the parental sets. It appears that both the pure parental mitogenomes and many combinations (if not all) of their genes including the chimeric genes can ensure mitochondrial functions in the alloploid nuclear background. The recessive nucleo-mitochondrial incompatibilities described previously in hybrids of these species did not directly cause hybrid sterility either. They were only manifested upon the loss of certain chromosomes in the nuclear genome (in alloaneuploid nuclear background)^21,33–35^. Such incompatibilities were also observed between haploid nuclei and allospecific mitochondria^60,61^. Our hybrids had complete alloploid karyotypes. The functionality of the chimeric mitogenomes further indicates that allospecific mitochondrial genes can also be compatible with each other. Although almost all hybrids were able to grow on a non-fermentable carbon source, their gene combinations may not support optimal mitochondrial performance (suboptimal nucleo-mitochondrial compatibility). In the literature, numerous examples of poor nucleo-mitochondrial synchronisation leading to some reduction of fitness were described (e.g.^62^). Epistatic interactions of genes of different descents within the chimeric genomes can also impair fitness. Wolters et al.^63^ found that mitochondrial alleles can interact epistatically (mito–mito epistasis) even within recombinant mtDNAs of *S*. *cerevisiae*. The potential impact of recombination on fitness can be investigated by examining, for example, respiratory efficiency, RPS-production and genome stability.

Here the stability of the recombinant genomes was investigated. All respiration-proficient hybrids produced non-respiring (*petite*) segregants, although the respiratory deficiency of certain hybrids was manifested only at 37 °C. In numerous cases, both non-ts and ts *petites* segregated from the same hybrid. Interestingly, there were also *petite* segregants for which 37 °C was restrictive even on the glucose-containing medium. The temperature-sensitive growth of these segregants might be attributed to LOH of nuclear genes, which are responsible for the different temperature sensitivity of the parents. It is well established that alloploid nuclear genomes occasionally lose chromosomes from one or the other parental subgenome during vegetative propagation (a phenomenon referred to as GARMI^44^). However, no change was detected in their karyotypes. The ability of all hybrids to produce spores at a permissive temperature also argues against drastic changes in ploidy (*MAT* heterozygosity required for sporulation was maintained). Nevertheless, the loss of respiration makes hybrids with recombinant mtDNA less competitive, and as a consequence, hybrids with chimeric mitogenomes can be outcompeted in mixed populations by strains of the parental species and/or by hybrids with pure parental mitogenomes. Thus, the recombination of the allospecific mtDNAs may (indirectly) contribute to the biological isolation of *S. cerevisiae* and *S. uvarum* by destabilising the mitogenome.

## Materials and methods

### Strains and culture media

The strains used in this study are listed in Table 1. YEA [0.5% (w/v) yeast extract, 2% (w/v) glucose and 2% (w/v)] agar plates were used for the maintenance of strains and for hybridisation. Hybrids were selected and maintained on SMA minimal medium [1% (w/v) glucose, 0.5% (w/v) (NH_4_)_2_SO_4_, 0.01% (w/v) KH_2_PO_4_, 0.005% (w/v) MgSO_4_, vitamins^64^ and 2% (w/v) agar]. Sporulation was tested on potassium-acetate medium [1% (w/v) potassium-acetate, 0.1% (w/v) yeast extract, 0.05% (w/v) glucose and 2% (w/v) agar]. Strains were tested for their ability to grow on a non-fermentable carbon source on YEAG plates [0.5% (w/v) yeast extract, 3% (v/v) glycerol and 2% (w/v) agar]. For DNA extraction, the strains were cultured in YEL [0.5% (w/v) yeast extract, and 2% (w/v) glucose] or YPD [1% (w/v) yeast extract, 2% (w/v) peptone and 2% (w/v) glucose].

### Hybridisation and hybrid selection

Hybridisation of auxotrophic S*. cerevisiae* and *S. uvarum* strains was performed as described previously^30^. Briefly, the auxotrophic strains were streaked on YEA plates. After 3 days of incubation the line-shaped cultures were replica-plated onto fresh YEA plates perpendicular to each other to produce grids of prints. After 5 days of incubation at 20 °C, the “grids” were replica-plated onto SMA plates on which the prints of the auxotrophic strains could not grow. Their hybrids appeared as prototrophic colonies at the intersections. Individual colonies were isolated as hybrids produced by different mating events and kept at -80 °C to prevent spontaneous segregation.

### Examination of phenotypic traits

The ability of strains to grow at 37 °C or to utilise glycerol as a carbon source was examined by a drop test: 10 μl of a 0.1 OD_590_ suspension was dropped on YEA and YEAG plates. Results were read after 6 days of incubation. Ascus formation was examined microscopically in cultures smeared onto potassium-acetate plates after 6 days of incubation at 25 °C. Melibiose fermentation was tested in Durham tubes in YEL medium containing melibiose instead of glucose. Bubble formation was visually monitored in the Durham tube at 25 °C for two weeks.

### Segregation test

The phenotypic stability of the hybrids with recombined mitochondrial genomes was tested by plating out cells on YEA plates. The size of the colonies was examined after seven days of incubation at 25 °C. For the hybrids, whose colonies were not uniform in size, small (putative *petites*) and large (*grandes*) colonies were isolated. Representatives of both size categories were tested for temperature sensitivity on YEA plates at 37 °C and for their ability to utilise glycerol as a carbon source on YEAG plates at 25 °C and 37 °C.

### Electrophoretic karyotyping

For karyotyping, overnight YEL cultures were used and the samples were prepared as described previously^20^. The chromosomal DNAs were separated with a CHEF-Mapper apparatus (Bio-Rad) in 1% (w/v) agarose gel (Chromosomal grade, Bio-Rad) prepared in 0.5 × TBE (45 mM Tris–borate, 1 mM EDTA).The running parameters were linear ramping from 40 to 120 s at 200 V for 24 h at 14 °C.

### mtDNA extraction and RFLP analysis

Total mitochondrial DNA was prepared from late stationary phase YEL cultures with the method described by Nguyen et al.^38^. The preparation was digested overnight with *Mbo*I and the fragments were separated by electrophoresis in 1.2% (w/v) agarose gel, with 1 × TBE.

### Genome sequencing and annotation

For DNA sequencing, a single colony from each strain was inoculated into liquid YPD medium and grown overnight at 28 °C. Samples of total cellular DNA were extracted essentially as described in^65^ and further purified using a DNeasy PowerClean Pro Cleanup kit (Qiagen). Paired-end (2 × 151) TruSeq PCR-free DNA libraries were prepared and sequenced on an Illumina NovaSeq 6000 platform at Macrogen Europe resulting in 30 to 48 million reads (4.6 to 7.3 Gbp per sample in total). The reads were trimmed and assembled using Trimmomatic (version 0.39^66^ in PE mode with the following settings ILLUMINACLIP:TruSeq3-PE.fa:2:30:10:2:keepBothReads LEADING:3 TRAILING:3 MINLEN:36) and SPAdes (version 3.15.4^67^ with—isolate option), respectively. The contigs containing mtDNA were identified in the assemblies using Megablast searches (BLAST + version 2.2.24^68^ with *S. cerevisiae* and *S. uvarum* mtDNA sequences as queries and further polished and the identified mtDNA contigs were extended by read mapping (in a custom mode with a minimum overlap of 50 bp and 100% sequence identity within the overlap) and circularised at overlapping terminal regions using Geneious (version 11.1.5; Biomatters). Final mtDNA sequences were annotated using the MFannot tool^69^ (with Yeast Mitochondrial genetic code option). The sequences were deposited in the GenBank database under the accession numbers listed in Supplementary Table 3S.The genetic maps were visualised using OGDraw^70^ and Proksee^71^ on-line tools and the figures were manually edited. To test the strains for homoplasmy, the trimmed Illumina reads (see above) were mapped to the assembled mtDNA sequences using BWA-MEM (version 0.7.18^72^) and the resulting alignments were filtered with SAMtools view (mapping quality ≥ 30; version 1.22^73^). Bases with a Phred quality below 20 (Q20) were masked using a custom script in Python (version 3.12.2). The alignments were visualized in JBrowse 2 (version 4.1.3^74^). Coverage and sequencing depth were calculated using SAMtools coverage (version 1.22^73^) (Supplementary Table 4S).

### Genome metrics and multiple sequence alignment

Overall genome relatedness indices were calculated in two ways. The average nucleotide identity (gANI) values were calculated with the algorithm developed by Goris et al.^75^ available at http://fbac.dmicrobe.cn/tools/ANI_calculator. ^76^ OrthoANIu (OrthoANI using USEARCH)^77^ values were calculated using the tool available at https://www.ezbiocloud.net/tools/ani. ^78^ ANI and OrthoANI measure nucleotide-level similarity between two genome sequences by fragmenting them and calculating average similarity values from fragment comparisons. Amino acid sequences were aligned with the Clustal W version 1.7^79^.

### Localisation of recombination sites

Sites of recombination were identified by aligning the recombinant genomes with the parental genomes using the Mauve algorithm^80^ available at https://darlinglab.org/mauve/mauve.html. Segments of identical sequences in the three genomes were considered recombination sites if they were flanked in the recombinant genome by different parental sequences. Blast similarity search in parental mitogenome sequences was performed with the tool available at https://blast.ncbi.nlm.nih.gov/Blast.cgi using default parameters.

## Supplementary Information

Below is the link to the electronic supplementary material.

Supplementary Material 1.

Supplementary Material 2.

## Data Availability

Original Illumina reads generated in this study were submitted to European Nucleotide Archive (ENA) database under the BioProject ID: PRJEB56422. The individual accession numbers are shown in Supplementary Table 3S. The genome sequence data are publically available in the NCBI Genome database under the accession numbers listed in the same table. Other data will be made available on request.
