# Supplementary material for: Nucleo-cytoplasmic compatibility in interspecies Saccharomyces hybrids and the destabilisation of the mitogenome by allospecific recombination
Source: Sci Rep. 2026 May 9;16:21279. doi: 10.1038/s41598-026-51924-x (PMC13346842; doi:10.1038/s41598-026-51924-x)
Supplement: Supplementary file 1 — Supplementary Material 1. [file 41598_2026_51924_MOESM1_ESM.pdf]

Supplementary Information

Nucleo-cytoplasmic compatibility in interspecies *Saccharomyces* hybrids and the destabilisation of the mitogenome by allospecific recombination

Zsuzsa Antunovics, Viktoria Hodorová, Jozef Nosek, Matthias Sipiczki

Supplementary Table 1S. Hybrids with recombinant mitotypes and their segregants: phenotypic and RFLP analysis. Strains with sequenced genomes are framed.

Strain/clone	Colony size[1] at 20 °C	Growth				Phenotype[4]	sporulation	RFLP[5]
		25 °C		37 °C				
		YEA[2]	YEAG[3]	YEA	YEAG			
**Parental strains**								
10-170	N	+	+	+	+	*grande*	no	c
10-522	N	+	+	-	-	ts *grande*	yes	u
**Hybrids and their (segregant) clones**								
B2	N	+	+	+	+	*grande*	yes	r
B2/1	small	+	+	+	+	*grande*	yes	r
B2/2	small	+	+	+	+	*grande*	yes	r
B2 p/1	small	+	-	+	-	*petite*		
B2 p/2	small	+	-	+	-	*petite*		
B2 p/3	small	+	-	+	-	*petite*		
B2 N/1	N	+	+	+	+	*grande*		
B2 N/2	N	+	+	+	-	ts *petite*		
B2 N/3	N	+	+	+	+	*grande*		
B4	N	+	+	+	-	ts *petite*	yes	r
B4/1	small	+	+	-	-	ts *grande*	yes	r
B4/2	small	+	-	-	-	*petite*, ts	no	s
B4 p/1	small	+	-	-	-	*petite*, ts		
B4 p/2	small	+	-	-	-	*petite*, ts		
B4 p/3	small	+	-	-	-	*petite*, ts		
B4 N/1	N	+	+	+	-	ts *petite*		
B4 N/2	N	+	+	+	-	ts *petite*		
B4 N/3	N	+	+	+	-	ts *petite*		
B11	N	+	+	+	+	*grande*	yes	r
B11/1	small	+	-	+	-	*petite*	yes	s
B11/2	small	+	+	+	+	*grande*	no	r
B11 p/1	small	+	-	+	-	*petite*		
B11 p/2	small	+	-	+	-	*petite*		
B11 p/3	small	+	-	+	-	*petite*		
B11 N/1	N	+	+	+	+	*grande*		
B11 N/2	N	+	+	+	+	*grande*		
B11 N/3	N	+	+	+	+	*grande*		
B16	N	+	+	+	+	*grande*	yes	r

B16/1	small	+	-	+	-	*petite*	no	s
B16/2	small	+	-	-	-	*petite*, ts	no	s
B16 p/1	small	+	+	+	+	*grande*		
B16 p/2	small	+	-	+	-	*petite*		
B16 p/3	small	+	-	+	-	*petite*		
B16 N/1	N	+	+	+	+	*grande*		
B16 N/2	N	+	+	+	+	*grande*		
B16 N/3	N	+	+	+	+	*grande*		
B20	N	+	+	+	+	*grande*	yes	r
B20/1	small	+	-	+	-	*petite*	no	r
B20/2	small	+	-	+	-	*petite*	no	s
B20 p/1	small	+	-	+	-	*petite*		
B20 p/2	small	+	-	+	-	*petite*		
B20 p/3	small	+	-	+	-	*petite*		
B20 N/1	N	+	+	+	+	*grande*		
B20 N/2	N	+	+	+	+	*grande*		
B20 N/3	N	+	+	+	+	*grande*		
B24	N	+	+	+	-	ts *petite*	yes	r
B24/1	small	+	-	-	-	*petite*, ts	no	s1
B24/2	small	+	-	-	-	*petite*, ts	no	s2
B24 p/1	small	+	-	+	-	*petite*		
B24 p/2	small	+	-	+	-	*petite*		
B24 p/3	small	+	-	+	-	*petite*		
B24 N/1	N	+	+	+	-	ts *petite*		
B24 N/2	N	+	+	+	-	ts *petite*		
B24 N/3	N	+	+	+	-	ts *petite*		
B25	N	+	+	+	+	*grande*	yes	r
B25/1	small	+	-	-	-	*petite*, ts	no	r
B25/2	small	+	-	-	-	*petite*, ts	no	s
B25 p/1	small	+	-	+	-	*petite*		
B25 p/2	small	+	-	+	-	*petite*		
B25 p/3	small	+	-	+	-	*petite*		
B25 N/1	N	+	+	+	+	*grande*		
B25 N/2	N	+	+	+	+	*grande*		
B25 N/3	N	+	+	+	+	*grande*		
B29	N	+	+	+	-	ts *petite*	yes	r
B29/1	small	+	-	-	-	*petite*, ts	no	s
B29/2	small	+	-	-	-	*petite*, ts	no	r
B29 p/1	small	+	-	-	-	*petite*, ts		
B29 p/2	small	+	-	-	-	*petite*, ts		
B29 p/3	small	+	-	-	-	*petite*, ts		
B29 N/1	N	+	+	+	-	ts *petite*		
B29 N/2	N	+	+	+	-	ts *petite*		
B29 N/3	N	+	+	+	-	ts *petite*		
B36	N	+	+	+	+	*grande*	yes	r
B36/1	small	+	+	+	+	*grande*	yes	r
B36/2	small	+	+	+	+	*grande*	yes	r

B36 p/1	small	+	-	-	-	*petite*, ts		
B36 p/2	small	+	-	-	-	*petite*, ts		
B36 p/3	small	+	-	-	-	*petite*, ts		
B36 N/1	N	+	+	+	+	*grande*		
B36 N/2	N	+	+	+	+	*grande*		
B36 N/3	N	+	+	+	+	*grande*		
B38	N	+	+	+	+	*grande*	yes	r
B38/1	small	+	-	-	-	*petite*, ts	no	s
B38/2	small	+	+	+	+	*grande*	yes	r
B38 p/1	small	+	+	+	+	*grande*		
B38 p/2	small	+	+	+	+	*grande*		
B38 p/3	small	+	-	-	-	*petite*, ts		
B38 N/1	N	+	+	+	+	*grande*		
B38 N/2	N	+	+	+	+	*grande*		
B38 N/3	N	+	+	+	+	*grande*		
B42	N	+	+	+	-	ts *petite*	yes	r
B42/1	small	+	+	+	-	ts *petite*	no	s1
B42/2	small	+	+	+	-	ts *petite*	no	s2
B42 p/1	small	+	-	+	-	*petite*		
B42 p/2	small	+	-	+	-	*petite*		
B42 p/3	small	+	+	+	-	ts *petite*		
B42 N/1	N	+	+	+	-	ts *petite*		
B42 N/2	N	+	+	+	-	ts *petite*		
B42 N/3	N	+	+	+	-	ts *petite*		
B53	N	+	+	+	-	ts *petite*	yes	r
B53/1	small	+	-	-	-	*petite*, ts	no	s
B53/2	small	+	+	+	-	ts *petite*	yes	r
B53 p/1	small	+	-	-	-	*petite*, ts		
B53 p/2	small	+	-	-	-	*petite*, ts		
B53 p/3	small	+	+	+	-	ts *petite*		
B53 N/1	N	+	+	+	-	ts *petite*		
B53 N/2	N	+	+	+	-	ts *petite*		
B53 N/3	N	+	+	+	-	ts *petite*		
B92	N	+	+	+	+	*grande*	yes	r
B92/1	small	+	-	-	-	*petite*, ts	no	r
B92/2	small	+	-	-	-	*petite*, ts	no	r
B92 p/1	small	+	-	+	-	*petite*		
B92 p/2	small	+	-	+	-	*petite*		
B92 p/3	small	+	-	+	-	*petite*		
B92 N/1	N	+	+	+	+	*grande*		
B92 N/2	N	+	+	+	+	*grande*		
B92 N/3	N	+	+	+	+	*grande*		
B93	N	+	+	+	+	*grande*	yes	r
B93/1	small	+	+	+	+	*grande*	yes	s1
B93/2	small	+	-	-	-	*petite*, ts	no	s2
B98	N	+	+	+	+	*grande*	yes	r

B98/1	small	+	-	-	-	*petite*, ts	no	s1
B98/2	small	+	-	-	-	*petite*, ts	no	s2
B98 p/1	small	+	+	+	+	grande		
B98 p/2	small	+	-	+	-	*petite*		
B98 p/3	small	+	-	+	-	*petite*		
B98 N/1	N	+	+	+	+	*grande*		
B98 N/2	N	+	+	+	+	*grande*		
B98 N/3	N	+	+	+	+	*grande*		
S2	N	+	+	+	+	grande	yes	
H1 S2p/1	small	+	-	-	-	*petite*, ts		
H1 S2p/2	small	+	-	-	-	*petite*, ts		
H1 S2p/3	small	+	+	+	+	*grande*		
H1 S2N/1	N	+	+	+	+	*grande*		
H1 S2N/1	N	+	+	+	+	*grande*		
H1 S2N/1	N	+	+	+	+	*grande*		
S6	N	+	+	+	+	*grande*	yes	
H2 S6p/1	small	+	-	-	-	*petite*, ts		
H2 S6p/2	small	+	-	-	-	*petite*, ts		
H2 S6p/3	small	+	-	-	-	*petite*, ts		
H2 S6N/1	N	+	+	+	+	*grande*		
H2 S6N/2	N	+	+	+	+	*grande*		
H2 S6N/3	N	+	+	+	+	*grande*		
S15	N	+	+	+	+	*grande*	yes	
H3 S15p/1	small	+	+	+	-	ts *petite*		
H3 S15p/2	small	+	+	+	-	ts *petite*		
H3 S15p/3	small	+	-	-	-	*petite*, ts		
H3 S15N/1	N	+	+	+	+	*grande*		
H3 S15N/2	N	+	+	+	+	*grande*		
H3 S15N/3	N	+	+	+	+	*grande*		
S24	N	+	+	+	-	ts *petite*	yes	
H4 S24p/1	small	+	-	+	-	*petite*		
H4 S24p/2	small	+	-	-	-	*petite*, ts		
H4 S24p/3	small	+	+	-	-	ts *grande*		
H4 S24N/1	N	+	+	+	-	ts *petite*		
H4 S24N/2	N	+	+	+	-	ts *petite*		
H4 S24N/3	N	+	+	+	-	ts *petite*		
A4	N	+	+	+	-	ts *petite*	yes	
A 4p/1	small	+	-	-	-	*petite*, ts		
A 4p/2	small	+	+	+	-	ts *petite*		
A 4p/3	small	+	-	-	-	*petite*, ts		
A 4N/1	N	+	+	+	-	ts *petite*		
A 4N/2	N	+	+	+	-	ts *petite*		
A 4N/3	N	+	+	+	-	ts *petite*		
A27	N	+	-	+	-	*petite*	no	
A 27p/1	small	+	-	+	-	*petite*		
A 27p/2	small	+	-	-	-	*petite*, ts		

A 27p!3	small	+	-	-	-	*petite*, ts		
A 27N/1	N	+	-	+	-	*petite*		
A 27N/2	N	+	-	+	-	petite		
A 27N/3	N	+	-	+	-	petite		
A33	N	+	+	+	-	ts *petite*	yes	
A 33p/1	small	+	-	-	-	*petite*, ts		
A 33p/2	small	+	-	-	-	*petite*, ts		
A 33p/3	small	+	-	-	-	*petite*, ts		
A 33N/1	N	+	+	+	-	ts *petite*		
A 33N/2	N	+	+	+	-	ts *petite*		
A 33N/3	N	+	+	+	-	ts *petite*		
A35	N	+	+	+	-	ts *petite*	yes	
A 35p/1	small	+	+	+	-	ts *petite*		
A 35p/2	small	+	-	-	-	*petite*, ts		
A 35p/3	small	+	-	-	-	*petite*, ts		
A 35N/1	N	+	+	+	-	ts *petite*		
A 35N/2	N	+	+	+	-	ts *petite*		
A 35N/3	N	+	+	+	-	ts *petite*		
A36	N	+	+	+	-	ts *petite*	yes	
A 36p/1	small	+	-	-	-	*petite*, ts		
A 36p/2	small	+	-	-	-	*petite*, ts		
A 36p/3	small	+	-	-	-	*petite*, ts		
A 36N/1	N	+	+	+	-	ts *petite*		
A 36N/2	N	+	+	+	-	ts petite		
A 36N/3	N	+	+	+	-	ts *petite*		
A37	N	+	+	+	-	ts *petite*	yes	
A 37p/1	small	+	-	-	-	*petite*, ts		
A 37p/2	small	+	-	-	-	*petite*, ts		
A 37p/3	small	+	-	-	-	*petite*, ts		
A 37N/1	N	+	+	+	-	ts *petite*		
A 37N/2	N	+	+	+	-	ts *petite*		
A 37N/3	N	+	+	+	-	ts *petite*		
A38	N	+	+	+	+	grande	yes	
A 38p/1	small	+	-	+	-	*petite*		
A 38p/2	small	+	-	+	-	*petite*		
A 38p/3	small	+	-	+	-	*petite*		
A 38N/1	N	+	+	+	+	*grande*		
A 38N/2	N	+	+	+	+	*grande*		
A 38N/3	N	+	+	+	+	*grande*		
A39	N	+	+	+	-	ts *petite*	yes	
A 39p/1	small	+	-	-	-	*petite*, ts		
A 39p/2	small	+	-	-	-	*petite*, ts		
A 39p/3	small	+	+	-	-	ts *grande*		
A 39N/1	N	+	+	+	-	ts *petite*		
A 39N/2	N	+	+	+	-	ts *petite*		
A 39N/3	N	+	+	+	-	ts *petite*		

Cu4,1	N	+	+	+	-	ts *petite*	yes	
Cu 4.1p/1	small	+	-	-	-	*petite*, ts		
Cu 4.1p/2	small	+	-	-	-	*petite*, ts		
Cu 4.1p/3	small	+	-	-	-	*petite*, ts		
Cu 4.1N/1	N	+	+	+	-	ts *petite*		
Cu 4.1N/2	N	+	+	+	-	ts *petite*		
Cu 4.1N/3	N	+	+	+	-	ts *petite*		
Cu4.8	N	+	+	+	-	ts *petite*	yes	
Cu 4.8p/1	small	+	-	+	-	*petite*		
Cu 4.8p/2	small	+	-	+	-	*petite*		
Cu 4.8p/3	small	+	-	+	-	*petite*		
Cu 4.8N/1	N	+	-	+	-	*petite*		
Cu 4.8N/2	N	+	+	+	-	ts *petite*		
Cu 4.8N/3	N	+	-	+	-	*petite*		

[1]N: normal-size (*grande*) colony; small: small (*petite*) colony
[2]YEA: yeast-extract-glucose-agar medium
[3]YEAG: yeast-extract-glycerol-agar medium
[4]Phenotypes:

grande: growth on glycerol at both temperatures

ts *grande* (*temperature-sensitive grande):* growth on glycerol at 25 °C but no growth at 37°C

petite: no growth on glycerol

ts *petite (temperature-sensitive petite)*: growth on glycerol at 25 °C but not at 37 °C

petite, ts (*petite with temperature sensitive growth)*: no growth at 37 °C and on glycerol at 25 °C

[5]r: recombinant pattern of the hybrid; s: segregant pattern different from r; s1 and s2 segregant patterns differing from each other.

+: growth; -: no growth

Supplementary Table 2S. Locations and sequences of recombination sites

Site	Location			Sequence
	in/at gene	in parental genomes		(shared by the parental genomes)
		S. c.	*S. u.*	
1	*rn1*	627-769	649-791	TAAACGATCGAACAGGTTGATGTTGCAATATCATCTGATTAATTGTGGTTAGTAGTGAAAGACAAATCTGGTTTGCAGATAGCTGGTTTTCTATGAAATTATGTAAGTATAGCCTTTATAAATAATAATTATTATATAATAT
2	*rn1*	1550-1635	1530-1615	TGTATACCGTAATGTAGACCGACTCAGGTATGTAAGTAGAGAATATGAAGGTGAATTAGATAATTAAAGGGAAGGAACTCGGCAAA
3	*rn1*	164ž-1769	1626-1749	AAGTTAGTCAATAAAGAGTAATAAGAACAAAGTTGTACAACTGTTTACTAAAAACACCGCACTTTGCAGAAACGATAAGTTTAAGTATAAGGTGTGAACTCTGCTCCATGCTTAATATATAAAT
4	*rn1*	1777-1785	1757-1765	TTTAACGAT
5	*rn1*	1792-2038	1772-2018	ATTAAATTTAGGTAAATAGCAGCCTTATTATGAGGGTTATAATGTAGCGAAATTCCTTGGCCTATAATTGAGGTCCCGCATGAATGACGTAATGATACAACAACTGTCTCCCCTTTAAGCTAAGTGAAATTGAAATCGTAGTGAAGATGCTATGTACCTTCAGCAAGACGGAAAGACCCTATGCAGCTTTACTGTAATTAGATAGATCGAATTATTGTTTATTATATTCAGCATATTAAGTAATCCT
6	*rn1*	2246-2320	2215-2289	ATAAGAGACAATCTCTAATTGGTAGTTTTGATGGGGCGTCATTATCAGCAAAAGTATCTGAATAAGTCCATAAAT
7	*rn1*	2678-2718	2561-2601	ATTGTAAAGGTTATTGATAACGAATAAAAGTTACGCTAGGGat
8	*rn1*	3901-4117	2641-2857	TATGTTTGCCACCTCGATGTCGACTCAACATTTCCTCTTGGTTGTAAAAGCTAAGAAGGGTTTGACTGTTCGTCAATTAAAATGTTACGTGAGTTGGGTTAAATACGATGTGAATCAGTATGGTTCCTATCTGCTGAAGGAAATATTATCAAATTAAATCTCATTATTAGTACGCAAGGACCATAATGAATCAACCCATGGTGTATCTATTGATAAT
9	*rn1*	4153-4178	2895-2920	ATATATTATCTATATTAGTTTATATT
10	*rn1*	4272-4283	3010-3021	ATCTGATAATTT
11	*trnY(gta)* *trnN(gtt)*	13301-13369	7832-7900	ATATGTCCTTATAGCTTATCGGTTAAAGCATCTCACTGTTAATGAGAATAGATGGGTTCAATTCCTATT

12	*COX2*	15740-15822	8922-9004	TATGCATGTTATTTTCAGGATTCAGCAACACCAAATCAAGAAGGTATTTTAGAATTACATGATAATATTATGTTTTATTTATT
13	*COX2*	15824-15858	9006-9040	GTTATTTTAGGTTTAGTATCTTGAATGTTATATAC
14	*COX2*	15860-15866	9042-9048	ATTGTTA
15	*COX2*	16073-16098	9255-9280	GATTTTATTAATGATAGTGGTGAAAC
16	*trnV(tac)*	20669-20744	12776-12851	AAGGAGATTAGCTTAATTGGTATAGCATTCGTTTTACACACGAAAGATTATAGGTTCGAACCCTATATTTCCTAAATCTAGATATAATA
17	*COX3*	21437-21498	13344-13405	TTTGCATTATTATCATTAGCATTATCACTAGCATTAACAATGCATGGTTATATTGGTAATAT
18	*COX3*	21500-21540	13407-13447	AATATGGTATATTTAGCATTATTTGTATTATTAACAAGTTC
19	*COX3*	21599-21657	13506-13564	GCAGTAAGAAAAGGTATTAATTTAGGTTTCTTAATGTTTGTATTATCTGAAGTATTAAT
20	*COX3*	21839-21901	13746-13808	GGTAATAGAAATAAAGCTTTATCAGGTTTATTAATTACATTCTGATTAATTGTTATTTTTGTT
21	*COX3*	21917-21963	13824-13870	GAATATACTAATGCTGCATTCACTATCTCTGATGGTGTTTATGGTTC
22	*COX3*	22103-22131	14010-14038	CTACATGTTTTAGATGTTATCTGATTATT
23	*trnM(cat)*	28469-28551	27555-27637	TATATAAATGCAATATGATGTAATTGGTTAACATTTTAGGGTCATGACCTAATTATATACGTTCAAATCGTATTATTGCTAAT
24	*COX1* exon1	43833-43977	31477-31621	TAAAAATGGTACAAAGATGATTATATTCAACAAATGCAAAAGATATTGCAGTATTATATTTTATGTTAGCTATTTTTAGTGGTATGGCAGGAACAGCAATGTCTTTAATCATTAGATTAGAATTAGCTGCACCTGGTTCACAATA
25	*COX1* exon1	43985-43986	31629-31630	GG
26	*COX1* exon2 /exon4	50745-50767	32349-32371	AGTGTAGATTTAGCAATTTTTGC
27	*COX1* exon2 /exon4	50769-50962	32373-32566	TTACATTTAACATCAATTTCATCATTATTAGGTGCTATTAATTTCATTGTAACAACATTAAATATGAGAACAAATGGTATGACAATGCATAAATTACCATTATTTGTATGATCAATTTTC

				ATTACAGCGTTCTTATTATTATTATCATTACCTGTATTATCTGCTGGTATTACAATGTTATTATTAGATAGAAA
28	*COX1* exon3 /exon5	52166-52230	34370-34434	ATTGGATTATTAGGATTCTTAGTATGATCACATCATATGTATATTGTAGGATTAGATGCAGATCT
29	*COX1* exon3/intron3 (orf308) / exon5/intron5 (orf306)	52274-52319	34478-34523	ACAGGAATTAAAATTTTCTCATGATTAATAAATCCCTTTAGCAAGG
30	*COX1*intron3 (orf308) / intron5 (orf306)	52561-52572	34774-34785	TATTGTAAAACA
31	*COX1* intron4 /intron6	53993-54016	36125-36148	ATAGCTAACGGGGAAACTCTTATA
32	*COX1* exon6 /exon8	56447-56466	38465-38484	TTAGGTATTAATGGTATGCC
33	*ATP6*	58613-58696	40139-40222	GGTTTACAATCATCATTTATTGATTTAAGTTGTTTAAATTTAACAACATTTTCATTATATACTATTATTGTATTATTAGTTATT
34	*ATP9*	76935-77047	47692-47804	ATTATGCAATTAGTATTAGCAGCTAAATATATTGGAGCAGGTATCTCAACAATTGGTTTATTAGGAGCAGGTATTGGTATTGCTATCGTATTCGCAGCTTTAATTAATGGTGT

Supplementary Table 3S. Databank accession numbers of parental and recombinant mitochondrial genome sequences

**Strain**	**Bioproject ID**	**Study ID**	**Sample ID**	**BioSample ID**	**Run ID**	**Experiment ID**	**GenBank ID**
**Parental strains**							
S. cerevisiae 10-170	PRJEB56422	ERP141357	ERS13514293	SAMEA111420618	ERR10302132	ERX9836747	OP499836
S. uvarum 10-522	PRJEB56422	ERP141357	ERS13514294	SAMEA111420619	ERR10302133	ERX9836748	OP499835
**Hybrids**							
B2	PRJEB56422	ERP141357	ERS17574755	SAMEA115038967	ERR12370444	ERX11747165	PP024686
B4	PRJEB56422	ERP141357	ERS17574756	SAMEA115038968	ERR12370445	ERX11747166	PP024687
B11	PRJEB56422	ERP141357	ERS17574757	SAMEA115038969	ERR12370442	ERX11747163	PP024680

B16	PRJEB56422	ERP141357	ERS17574758	SAMEA115038970	ERR12370446	ERX11747167	PP024691
B20	PRJEB56422	ERP141357	ERS17574759	SAMEA115038971	ERR12370447	ERX11747168	PP024702
B24	PRJEB56422	ERP141357	ERS17574760	SAMEA115038972	ERR12370448	ERX11747169	PP024695
B25	PRJEB56422	ERP141357	ERS17574761	SAMEA115038973	ERR12370449	ERX11747170	PP024697
B29	PRJEB56422	ERP141357	ERS17574762	SAMEA115038974	ERR12370450	ERX11747171	PP024700
B36	PRJEB56422	ERP141357	ERS17574764	SAMEA115038976	ERR12370452	ERX11747173	PP024683
B38	PRJEB56422	ERP141357	ERS17574765	SAMEA115038977	ERR12370453	ERX11747174	PP024701
B42	PRJEB56422	ERP141357	ERS17574766	SAMEA115038978	ERR12370455	ERX11747176	PP024685
B53	PRJEB56422	ERP141357	ERS17574767	SAMEA115038979	ERR12370454	ERX11747175	PP024681
B92	PRJEB56422	ERP141357	ERS17574771	SAMEA115038983	ERR12370509	ERX11747194	PP024687
B93	PRJEB56422	ERP141357	ERS17574772	SAMEA115038984	ERR12370508	ERX11747193	PP024680
B98	PRJEB56422	ERP141357	ERS17574773	SAMEA115038985	ERR12370507	ERX11747192	PP024691
S2	PRJEB56422	ERP141357	ERS13514295	SAMEA111420620	ERR10302134	ERX9836749	OP499837
S6	PRJEB56422	ERP141357	ERS13514296	SAMEA111420621	ERR10302135	ERX9836750	OP499838
S15	PRJEB56422	ERP141357	ERS13514297	SAMEA111420622	ERR10302136	ERX9836751	OP499839
S24	PRJEB56422	ERP141357	ERS13514298	SAMEA111420623	ERR10302137	ERX9836752	OP499840
A4	PRJEB56422	ERP141357	ERS17574747	SAMEA115038959	ERR12370207	ERX11746928	PP024699
A27	PRJEB56422	ERP141357	ERS17574748	SAMEA115038960	ERR12370209	ERX11746930	PP024705
A33	PRJEB56422	ERP141357	ERS17574749	SAMEA115038961	ERR12370208	ERX11746929	PP024684
A35	PRJEB56422	ERP141357	ERS17574750	SAMEA115038962	ERR12370206	ERX11746927	PP024678
A36	PRJEB56422	ERP141357	ERS17574751	SAMEA115038963	ERR12370210	ERX11746931	PP024690
A37	PRJEB56422	ERP141357	ERS17574752	SAMEA115038964	ERR12370436	ERX11747157	PP024694
A38	PRJEB56422	ERP141357	ERS17574753	SAMEA115038965	ERR12370435	ERX11747156	PP024698
Cu4.1	PRJEB56422	ERP141357	ERS17574774	SAMEA115038986	ERR12370438	ERX11747159	PP024682
Cu4.8	PRJEB56422	ERP141357	ERS17574775	SAMEA115038987	ERR12370439	ERX11747160	PP024693

Supplementary Table 4S. Metrics generated by the SAMtools coverage tool (Li et al., 2009) from the reads aligned to the mtDNA sequences of parental and hybrid strains.

Strain	Start	End	Number of reads	Covbases	Coverage	Mean depth	Mean base quality	Mean map quality
**Parental strains**								
S. cerevisiae 10-170	1	88223	4112409	88223	100,0	6976,5	35,1	60,0
S. uvarum 10-522	1	64015	2608872	64015	100,0	6105,6	35,4	60,0
**Hybrids**								
B4	1	70767	2612046	70767	100,0	5521,1	34,9	60,0
B20	1	85599	2103575	85599	100,0	3669,5	34,7	59,9
B24	1	71295	2292284	71295	100,0	4817,1	35,1	60,0
B25	1	88569	2357492	88569	100,0	3983,1	35,0	60,0
B29	1	71198	2265707	71198	100,0	4766,3	35,0	60,0
B33	1	87461	2272624	87461	100,0	3884,8	35,0	59,9
B36	1	81390	2757440	81390	100,0	5064,3	34,9	59,9
B38	1	86948	2550891	86948	100,0	4385,9	35,0	59,9
B42	1	71373	3074564	71373	100,0	6452,9	35,0	60,0
B53	1	70695	2683623	70695	100,0	5683,2	34,9	60,0
B92	1	86948	2790068	86948	100,0	4794,2	34,8	59,9
B93	1	81392	2078841	81392	100,0	3813,9	34,8	59,9
B98	1	86948	2182517	86948	100,0	3740,9	34,6	59,9
S2	1	71373	2519502	71373	100,0	5284,9	35,0	60,0
S6	1	81419	2826067	81419	100,0	5195,3	35,2	59,9
S15	1	81390	3484244	81390	100,0	6408,2	35,2	60,0
S24	1	70695	3323341	70695	100,0	7047,7	35,2	60,0
A4	1	71337	2917395	71337	100,0	6102,4	34,8	60,0
A27	1	70686	3337958	69641	98,5	6907,1	34,7	59,9
A33	1	70784	3165609	70784	100,0	6661,3	34,7	60,0
A35	1	71198	3015600	71198	100,0	6326,3	35,0	60,0
A36	1	82142	4389712	82142	100,0	7977,1	34,8	60,0
A37	1	71373	2405306	71373	100,0	5030,2	34,9	60,0

A38	1	81390	3545346	81390	100,0	6491,1	34,7	59,9
B11	1	81388	3203046	81388	100,0	5888,9	35,0	60,0
B16	1	86948	3125024	86948	100,0	5369,7	34,7	59,9
B2	1	81390	2825788	81390	100,0	5193,2	34,9	60,0
Cu4.1	1	81590	2994977	81590	100,0	5466,7	34,7	59,9
Cu4.8	1	70684	2788232	70684	100,0	5881,1	34,8	60,0

Reference:

Li, H., et al. The Sequence Alignment/Map format and SAMtools. *Bioinformatics (Oxford, England).* **25**, 2078–2079 (2009). https://doi.org/10.1093/bioinformatics/btp352

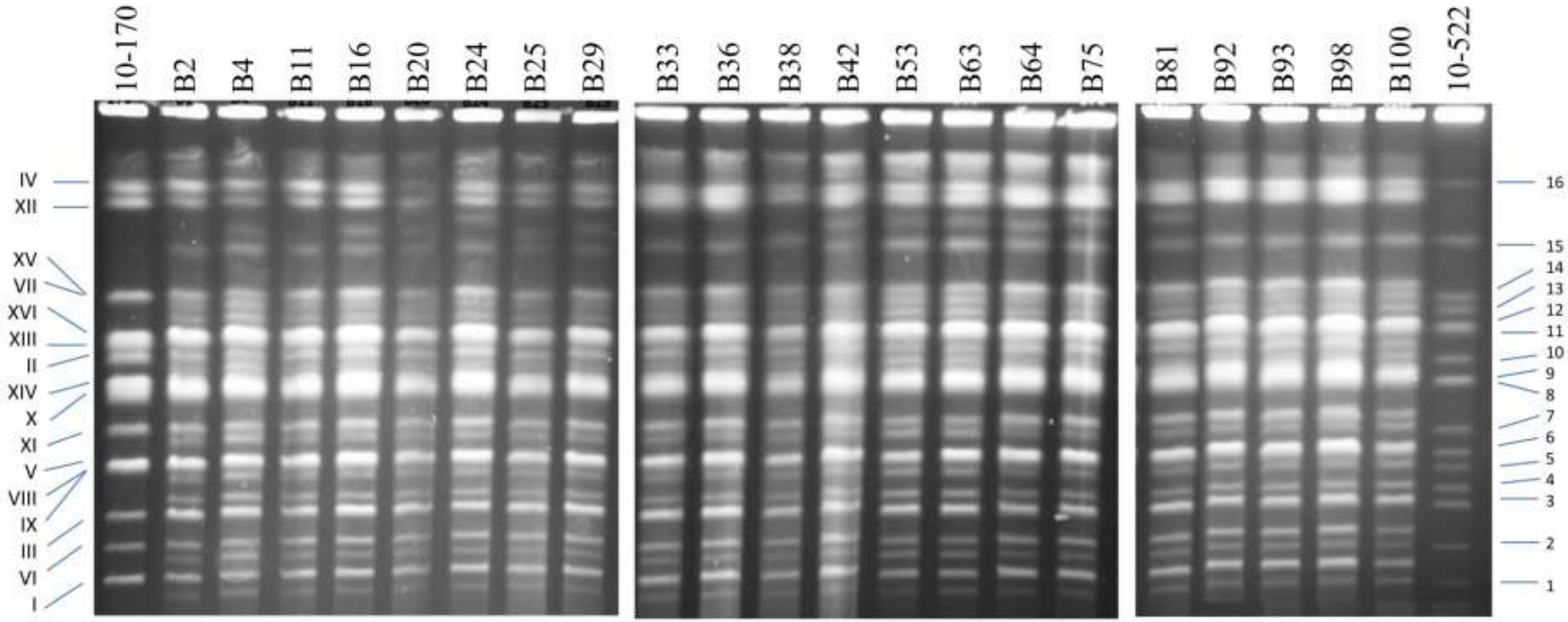

Supplementary Fig. 1S. Examples of electrophoretic karyotypes of hybrids from series B. The *S. uvarum* chromosomes are numbered according to Nguyen et al., 2000.

Reference:

Nguyen, H.V., Lepingle, A. & Gaillardin, C. Molecular typing demonstrates homogeneity of *Saccharomyces uvarum* strains and reveals the existence of hybrids between *S. uvarum* and *S. cerevisiae*, including the *S. bayanus* type strain CBS 380. *Syst. Appl. Microbiol.* **23**, 71–85 (2000).

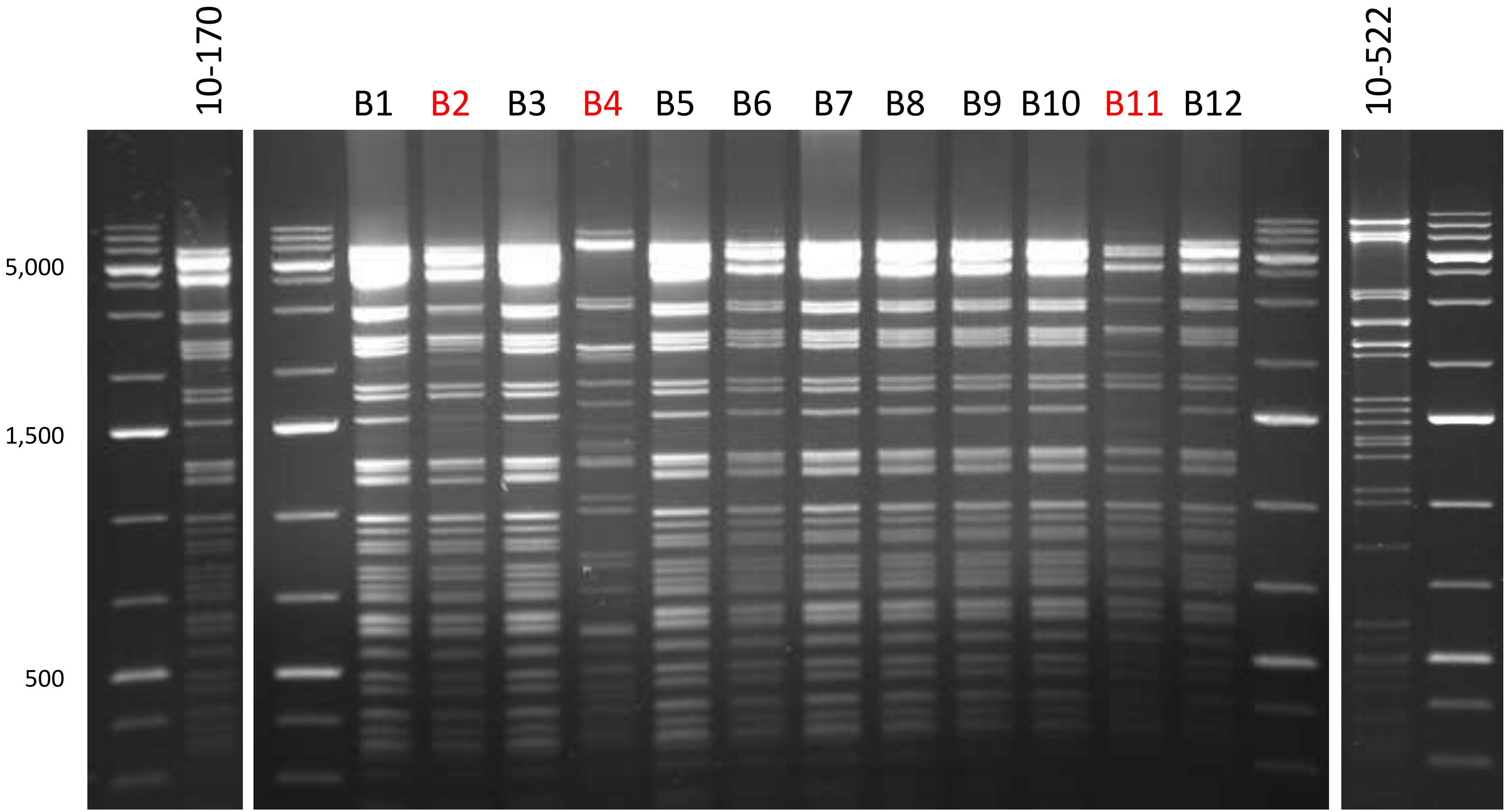

Supplementary Fig. 2S. Examples of RFLP patterns of mitogenomes of hybrids from series B. Recombinant patterns are marked with red colour.

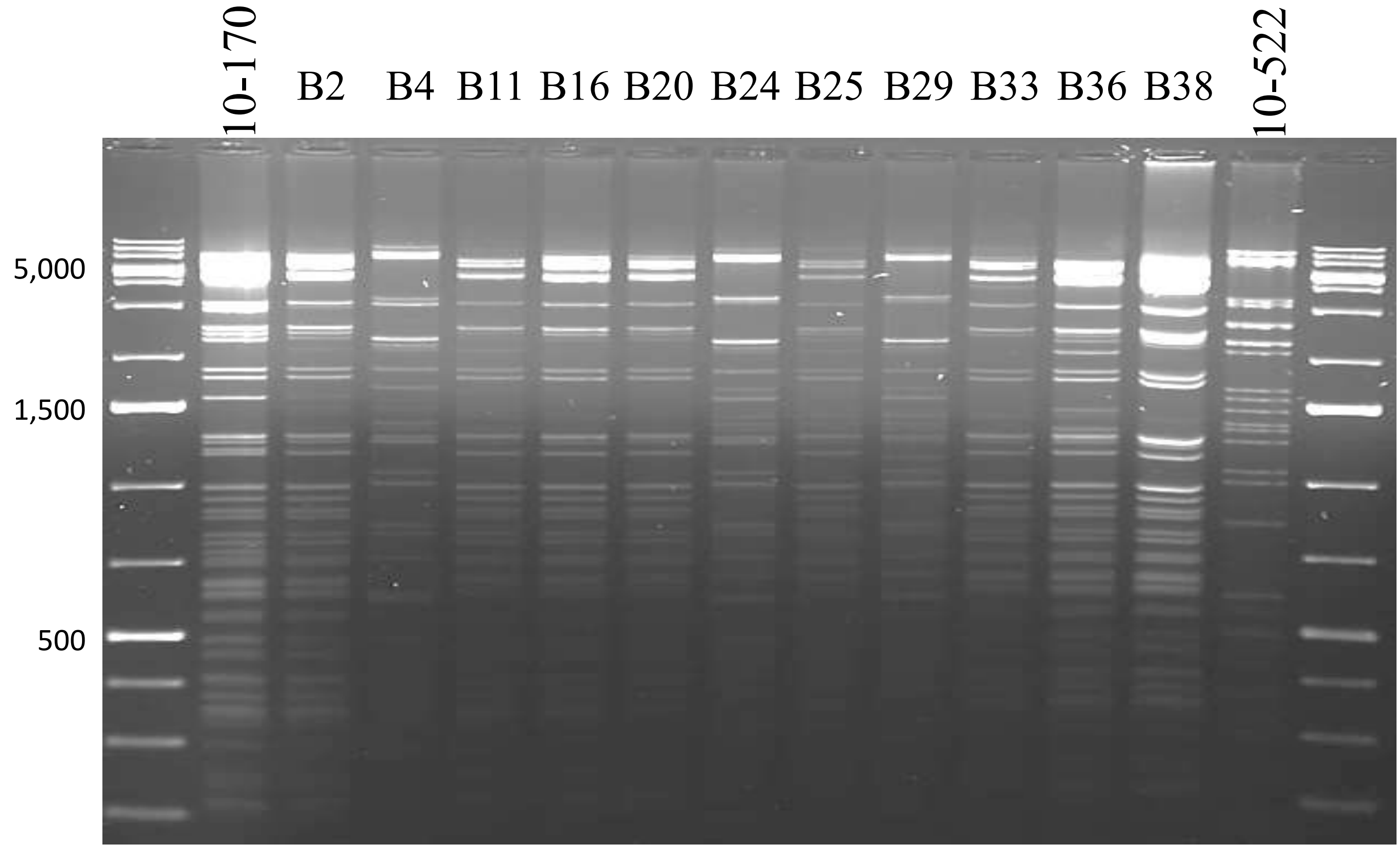

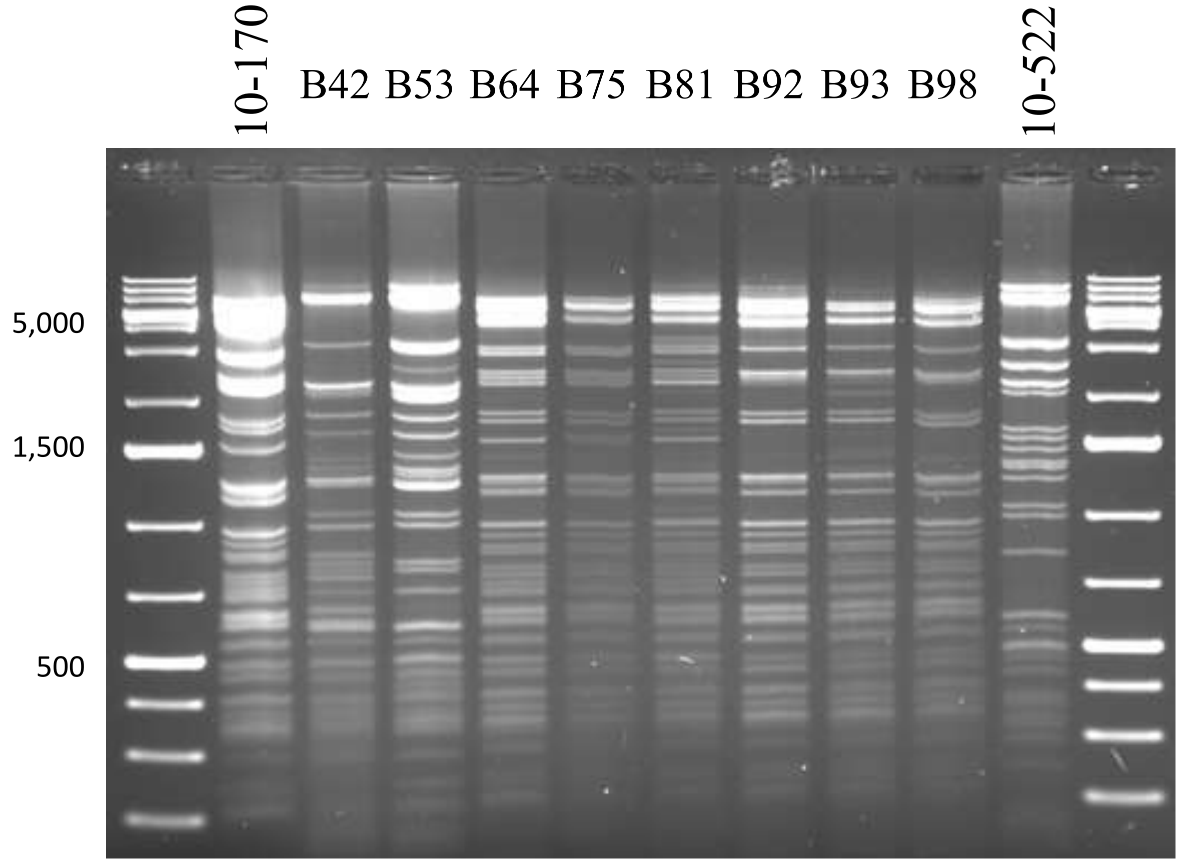

Supplementary Fig. 3S. Diversity of RFLP patterns of recombinant mitotypes of hybrids from series B

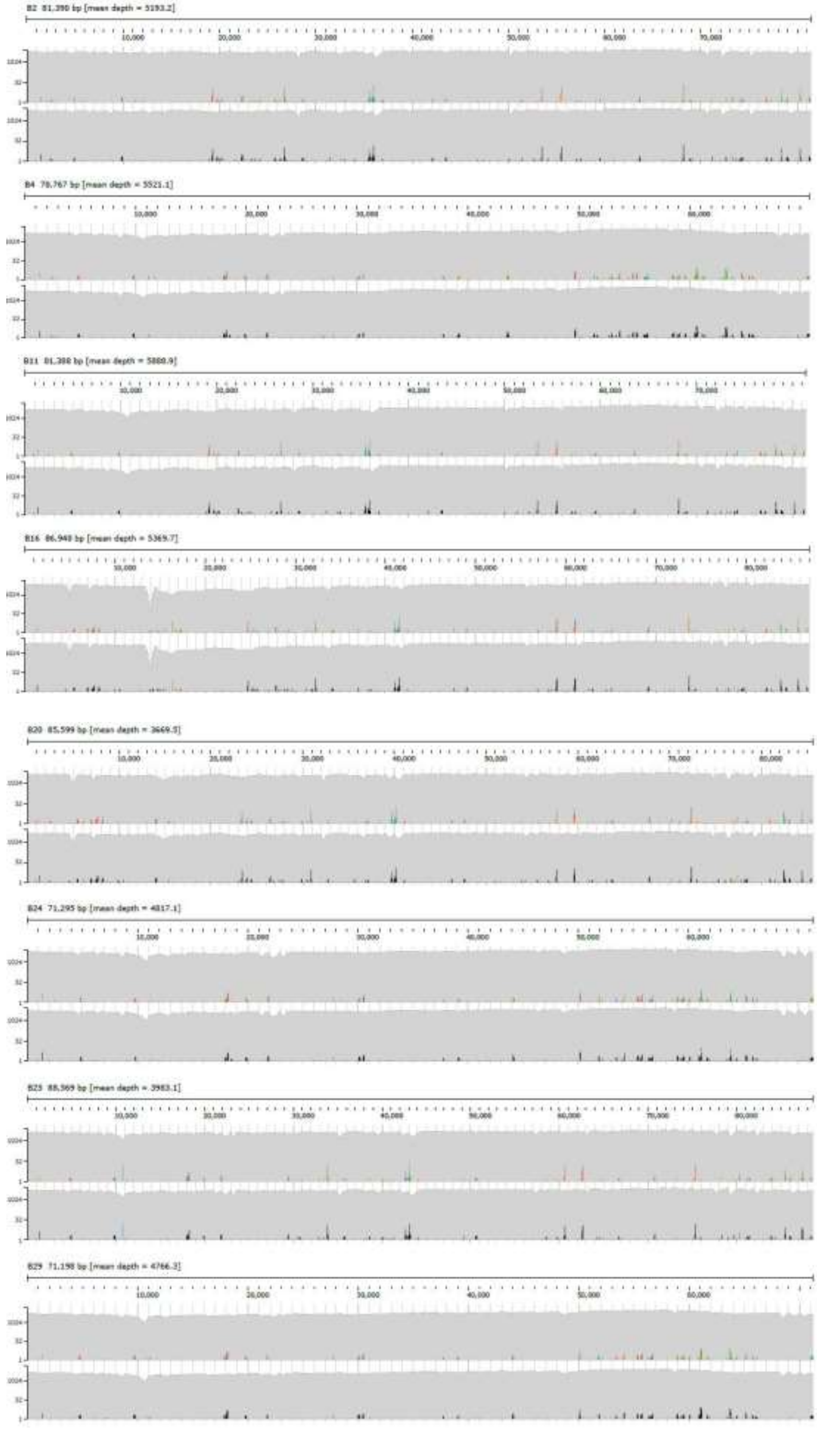

B2 81,390 bp [mean depth = 5193.2]
B4 78,767 bp [mean depth = 5521.1]
B11 81,388 bp [mean depth = 5880.9]
B16 86,948 bp [mean depth = 5369.7]
B20 85,599 bp [mean depth = 3669.5]
B24 71,295 bp [mean depth = 4817.1]
B25 88,369 bp [mean depth = 3983.1]
B29 71,198 bp [mean depth = 4766.3]

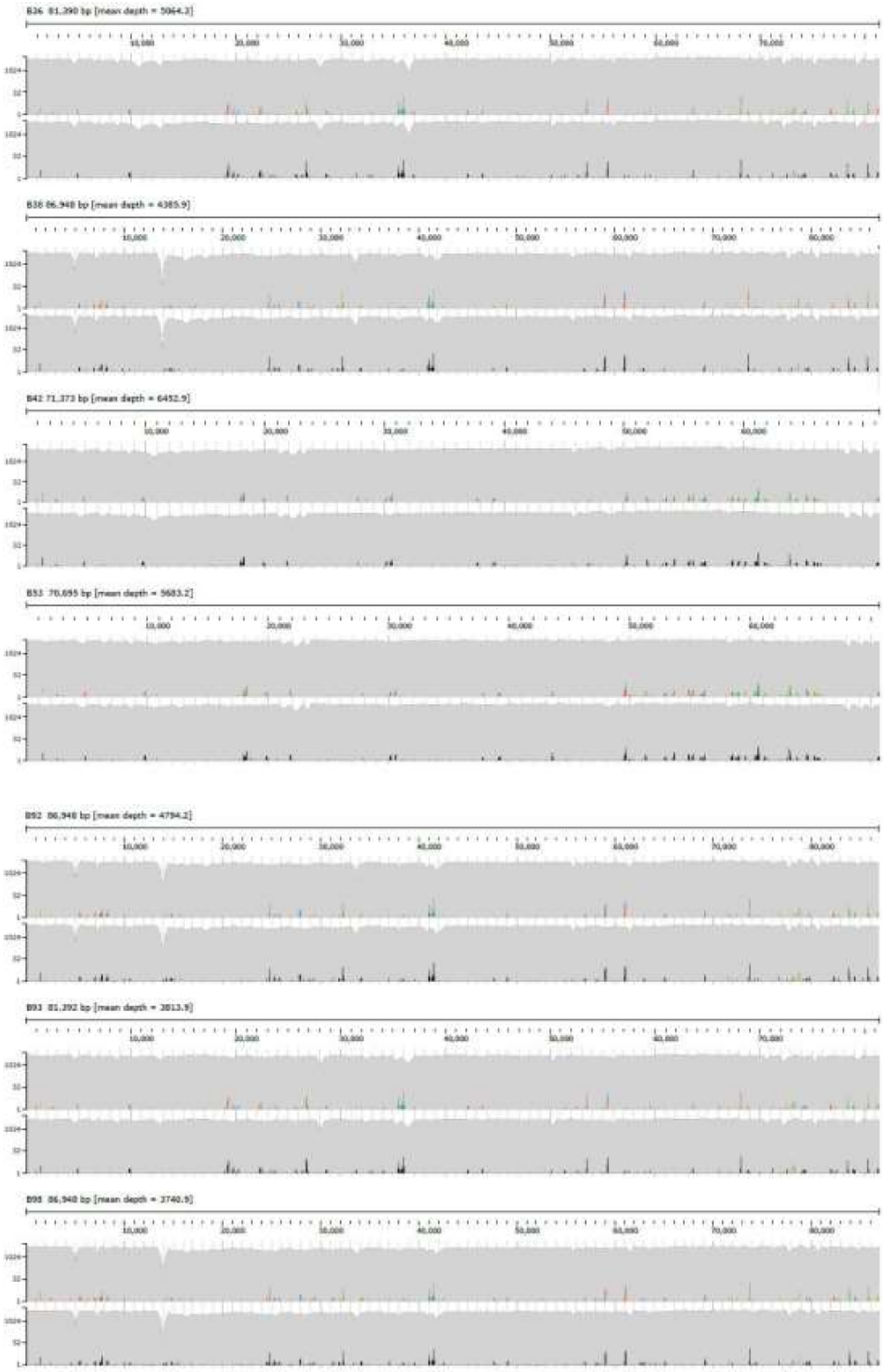
B36 81,390 bp [mean depth = 5064.2]
B38 86,948 bp [mean depth = 4385.9]
B42 71,373 bp [mean depth = 6452.9]
B53 70,695 bp [mean depth = 5683.2]
B92 86,948 bp [mean depth = 4794.2]
B93 81,392 bp [mean depth = 3813.9]
B98 86,948 bp [mean depth = 3740.9]

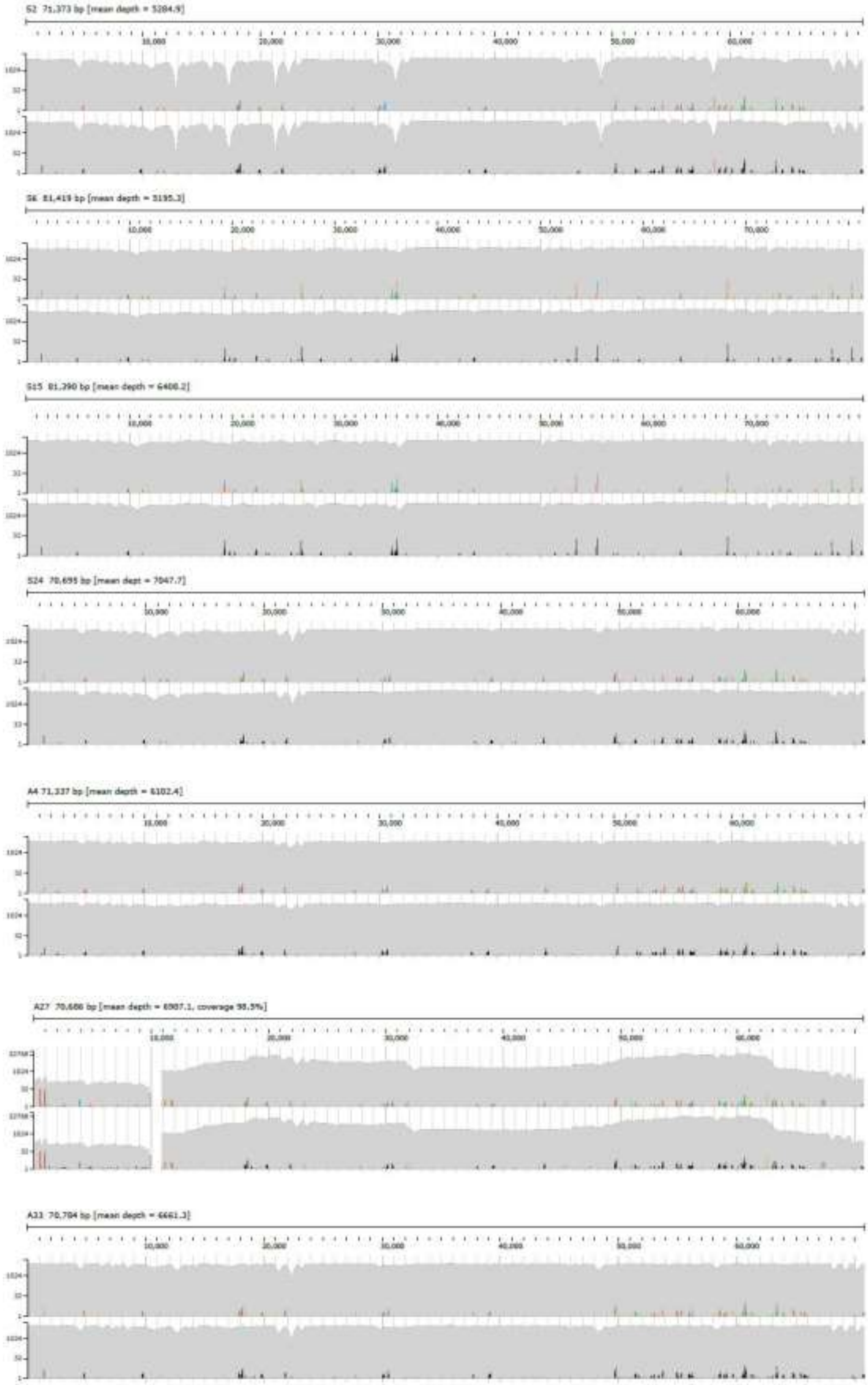
S2 71,373 bp [mean depth = 5284.9]
S6 81,419 bp [mean depth = 5195.3]
S15 81,390 bp [mean depth = 6400.2]
S24 70,695 bp [mean dept = 7047.7]
A4 71,337 bp [mean depth = 6102.4]
A27 70,686 bp [mean depth = 6907.1, coverage 98.5%]
A33 70,784 bp [mean depth = 6661.3]

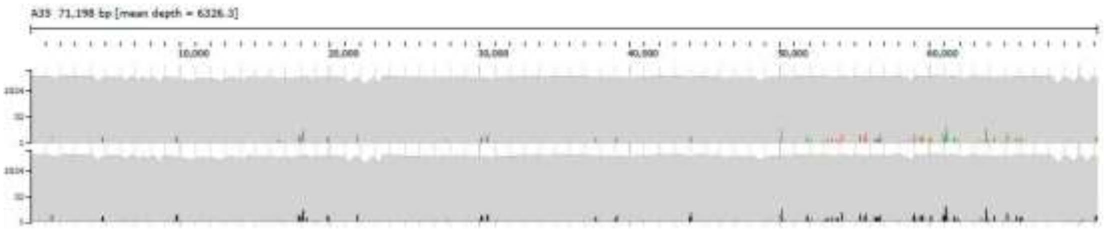
A35 71,198 bp [mean depth = 6326.3]

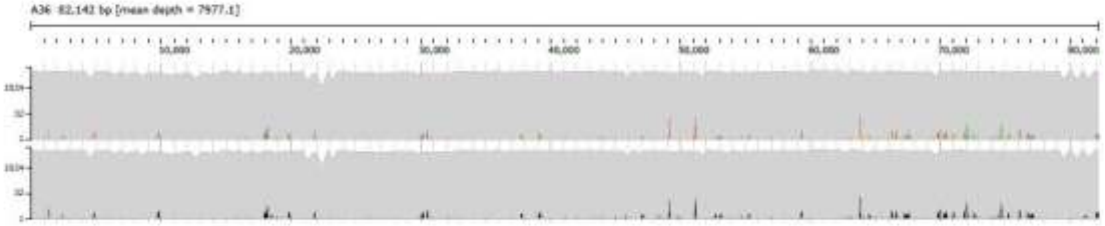
A36 82,142 bp [mean depth = 7977.1]

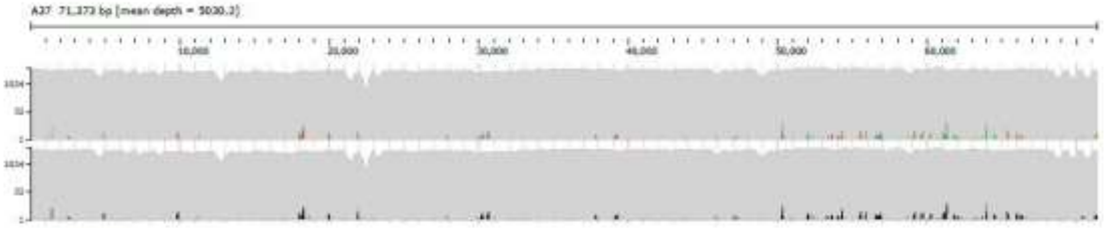
A37 71,373 bp [mean depth = 5030.2]

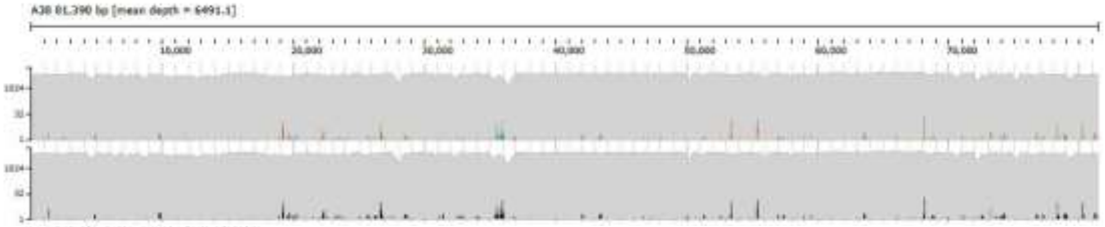
A38 81,390 bp [mean depth = 6491.1]

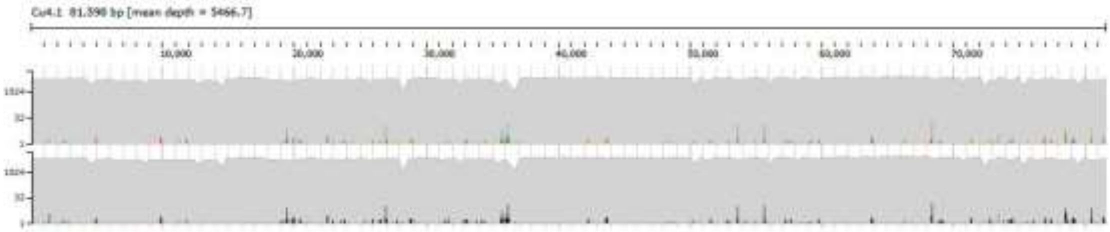
Cu4.1 81,398 bp [mean depth = 5466.7]

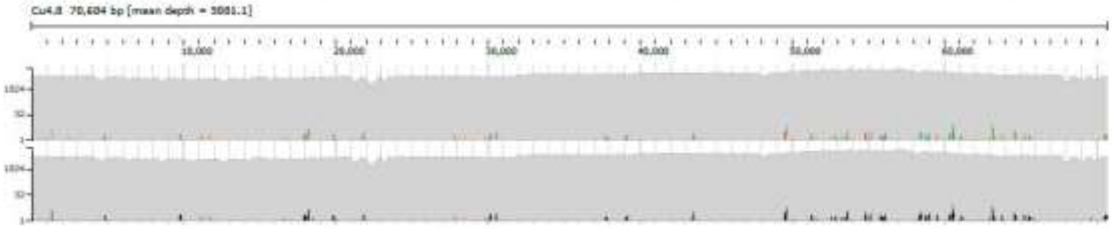
Cu4.8 70,604 bp [mean depth = 5081.1]

Parents:

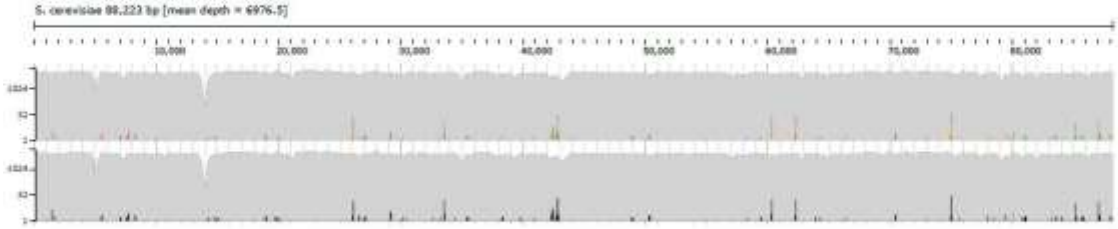
S. cerevisiae 88,223 bp [mean depth = 6976.5]

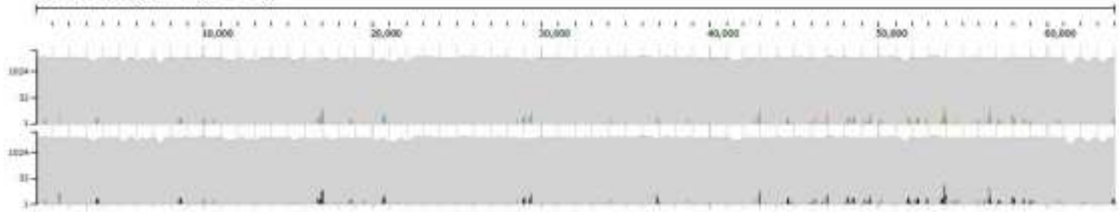
S. ovarum 64,015 bp [mean depth = 6105.6]

Supplementary Fig. 4S. The coverage of mtDNA sequences by Illumina reads visualized in JBrowse 2. The variant bases are coloured: A – green, C – blue, G – orange, T – red. In the lower panel, the low-quality bases (< Q20) were masked in black. For each strain, the size of mtDNA (in base pairs) and mean depth values are shown.

Strain	Size of mtDNA (bp)	OrthoANIu values with		gANI values with		Aligned fractions of the genomes			
		S. c. 10-170	*S. u.* 10-522	*S. c.* 10-170	*S. u.* 10-522	*S. c.* - rec	rec - *S.c.*	*S. u.* - rec	rec - *S. u.*
S. cerevisiae 10-170	88223	100.00	79.30	100.00	95.11	0.26[1]	0.35[2]		
S. uvarum 10-522	64015	79.30	100.00	95.11	100.00				
S2	71373	91.93	97.71	98.89	96.62	0.68	0.68	0.70	0.51
S6	81419	99.47	88.94	99.94	98.97	0.93	0.85	0.45	0.31
S15	81390	99.53	88.65	99.93	96.68	0.94	0.88	0.44	0.30
S24	70695	89.32	99.40	98.14	99.88	0.63	0.64	0.75	0.56
A4	71337	88.31	98.98	98.13	99.89	0.63	0.58	0.85	0.58
A27	70686	89.51	99.40	98.29	99.71	0.64	0.64	0.76	0.55
A33	70784	91.00	99.15	98.80	99.04	0.65	0.65	0.69	0.51
A35	71198	89.93	99.85	98.03	99.93	0.61	0.57	0.83	0.58
A36	82142	97.10	97.54	99.70	98.30	0.82	0.72	0.60	0.39
A37	71373	91.90	97.72	98.89	98.97	0.68	0.68	0.70	0.51
A38	81390	99.52	88.66	99.93	96.48	0.94	0.88	0.44	0.30
Cu4.1	81590	99.75	87.92	99.94	96.62	0.93	0.86	0.45	0.31
Cu4.8	70684	89.05	99.59	98.16	99.88	0.62	0.65	0.73	0.56
B2	81390	99.48	88.76	99.93	96.58	0.94	0.87	0.45	0.30
B4	70767	89.73	99.04	98.36	99.48	0.64	0.63	0.75	0.54
B11	81388	99.49	88.75	99.93	96.58	0.94	0.86	0.45	0.30
B16	86948	98.99	84.70	100.00	96.16	0.94	0.88	0.42	0.29
B20	85599	99.02	85.63	99.96	96.19	0.93	0.88	0.41	0.28
B24	71295	89.64	99.14	98.13	99.89	0.63	0.58	0.85	0.58
B25	88569	99.36	94.85	99.93	97.92	0.90	0.80	0.53	0.34
B29	71198	89.94	99.85	98.04	99.95	0.61	0.58	0.83	0.58
B36	81390	99.51	88.68	99.93	96.58	0.94	0.87	0.45	0.30
B38	86948	99.00	84.70	100.00	95.94	0.97	0.92	0.42	0.29
B42	71373	91.89	97.73	98.89	98.97	0.68	0.68	0.70	0.51
B53	70695	89.31	99.42	98.14	99.88	0.63	0.64	0.75	0.56
B92	86948	99.03	84.47	100.00	95.49	0.94	0.88	0.42	0.29
B93	81392	99.45	88.84	99.94	96.72	0.93	0.85	0.47	0.31
B98	86948	99.01	84.56	99.99	95.94	0.94	0.86	0.41	0.27
Average of hybrids		94.97	93.31	99.21	97.97				

ANI values: 79 100

Supplementary Fig. 5S. Genomic metrics. Size of genomes and similarity values between parental mitogenomes and recombinant mitogenomes calculated by OrthoANIu and gANI. [1] *S. cerevisiae* 10-170 compared to *S. uvarum* 10-522. [2] *S. uvarum* 10-522 compared to *S. cerevisiae* 10-170. Both ANI and OrthoANI are computational metrics used to measure nucleotide-level similarity between two genome sequences. Both calculate mean sequence identity of fragments after fragmentation of both genome sequences. ANI calculates it from all compared fragment pairs, while OrthoANI considers only orthologous fragment pairs (reciprocal best-hit pairs).

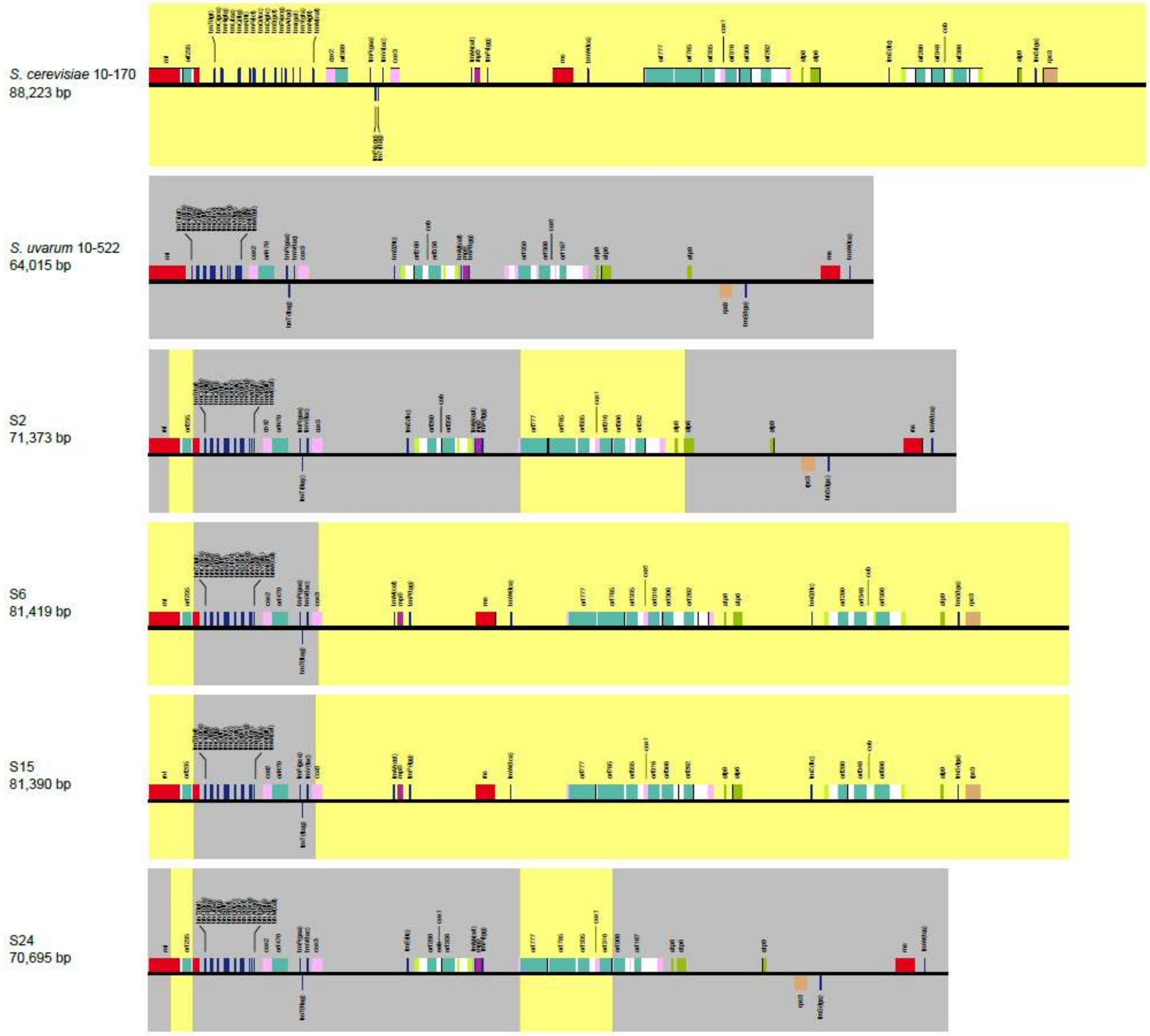
S. cerevisiae 10-170
88,223 bp
S. uvarum 10-522
64,015 bp
S2
71,373 bp
S6
81,419 bp
S15
81,390 bp
S24
70,695 bp

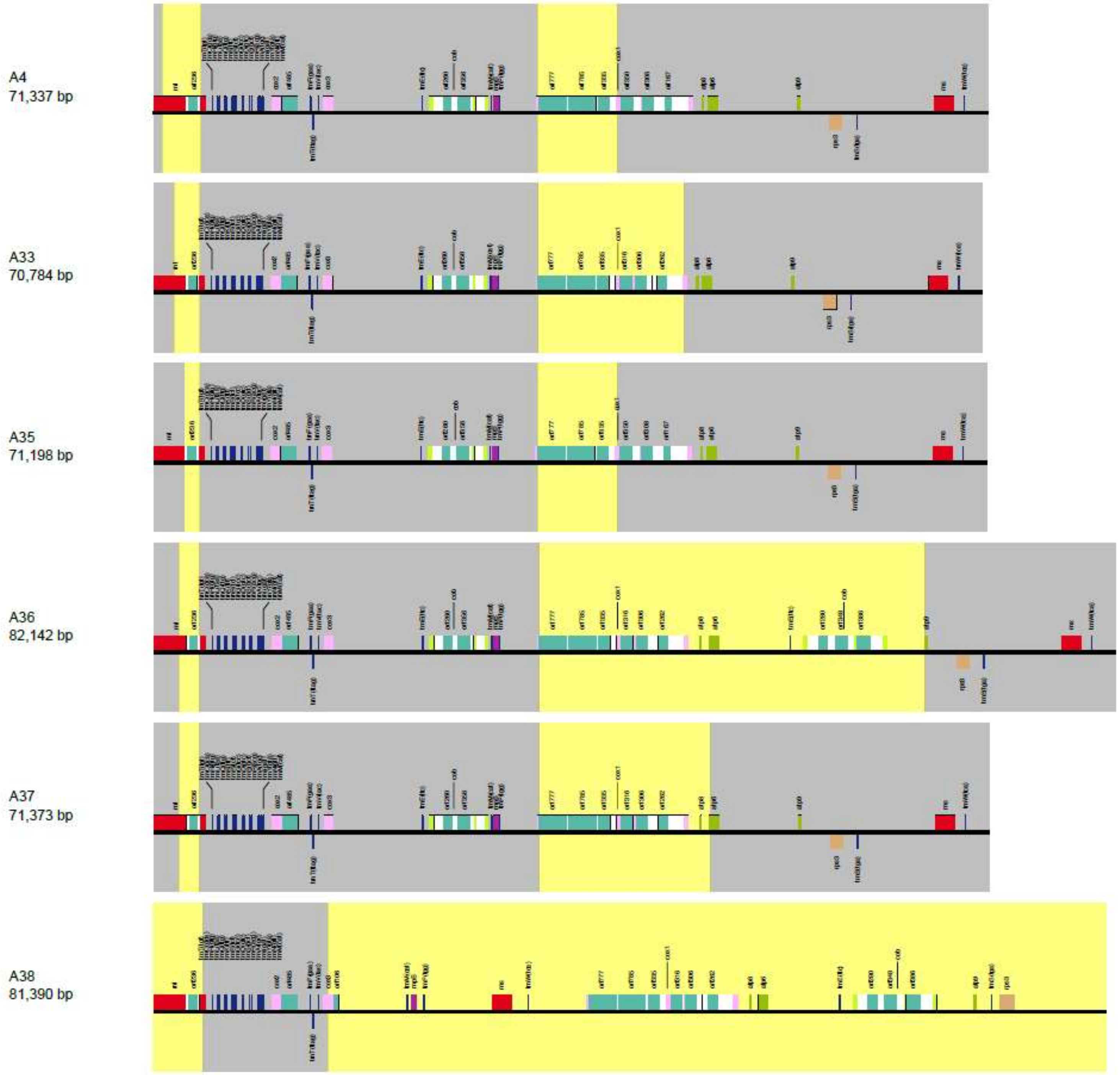
A4
71,337 bp
A33
70,784 bp
A35
71,198 bp
A36
82,142 bp
A37
71,373 bp
A38
81,390 bp

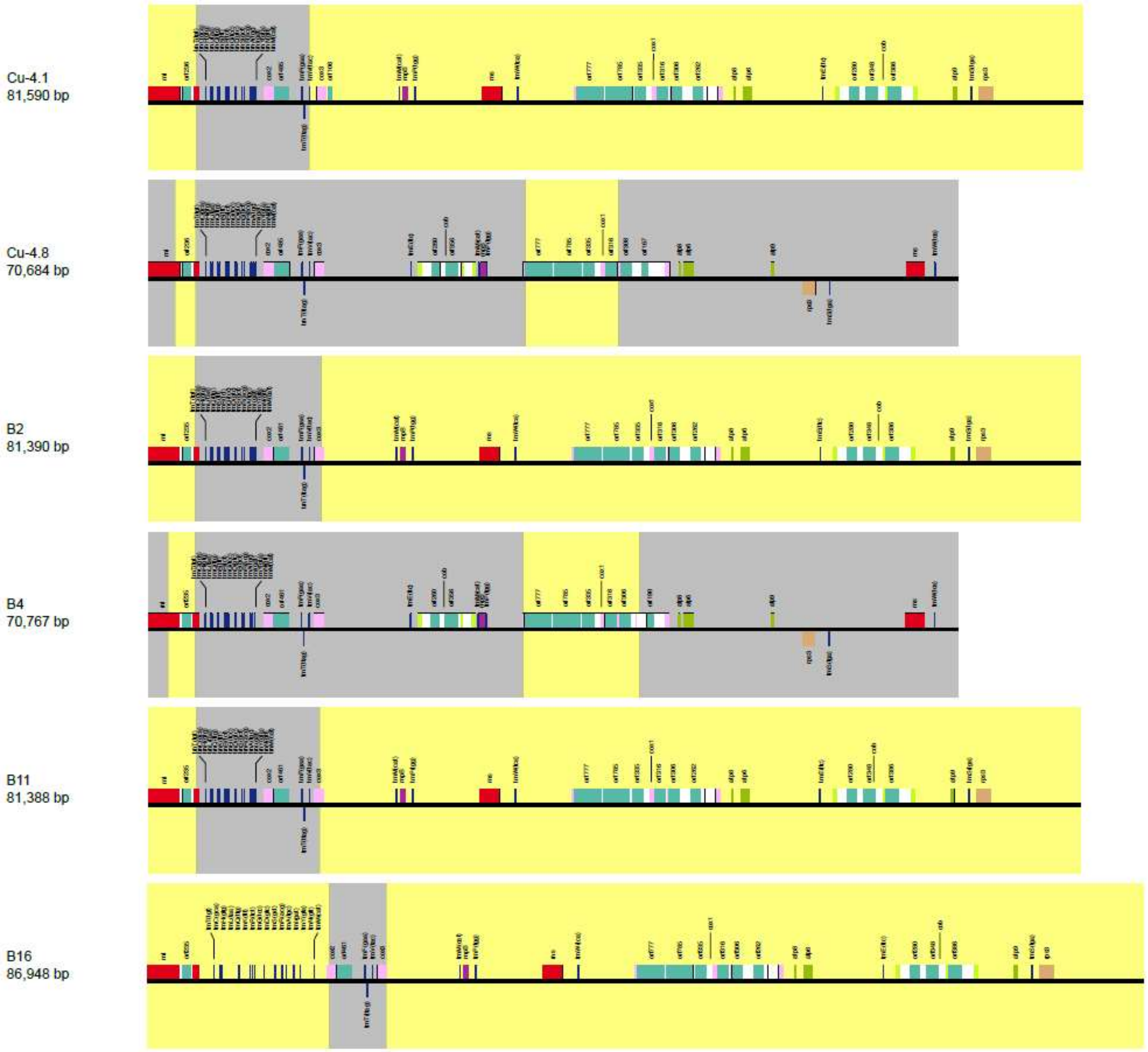

Cu-4.1
81,590 bp
Cu-4.8
70,684 bp
B2
81,390 bp
B4
70,767 bp
B11
81,388 bp
B16
86,948 bp

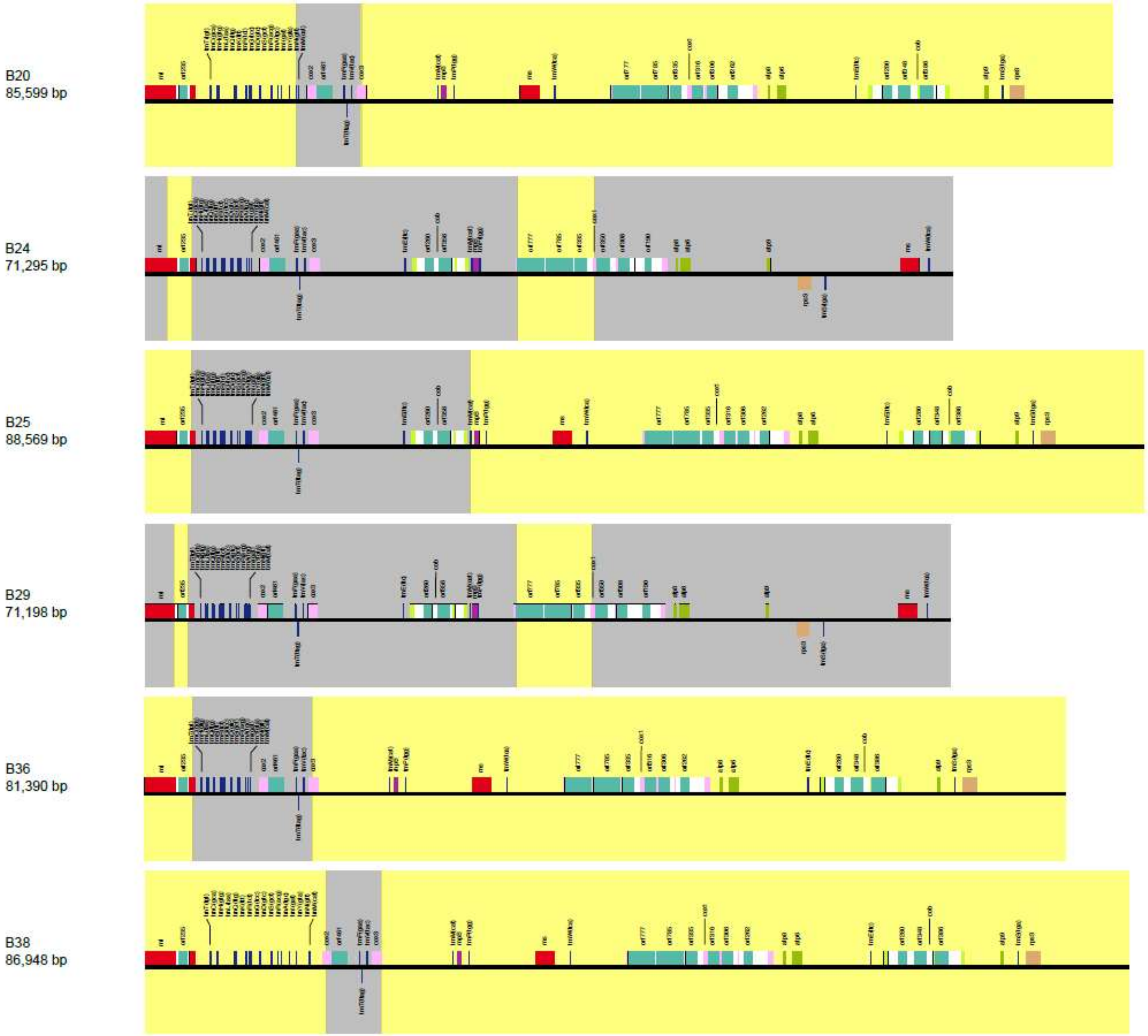
B20
85,599 bp
B24
71,295 bp
B25
88,569 bp
B29
71,198 bp
B36
81,390 bp
B38
86,948 bp

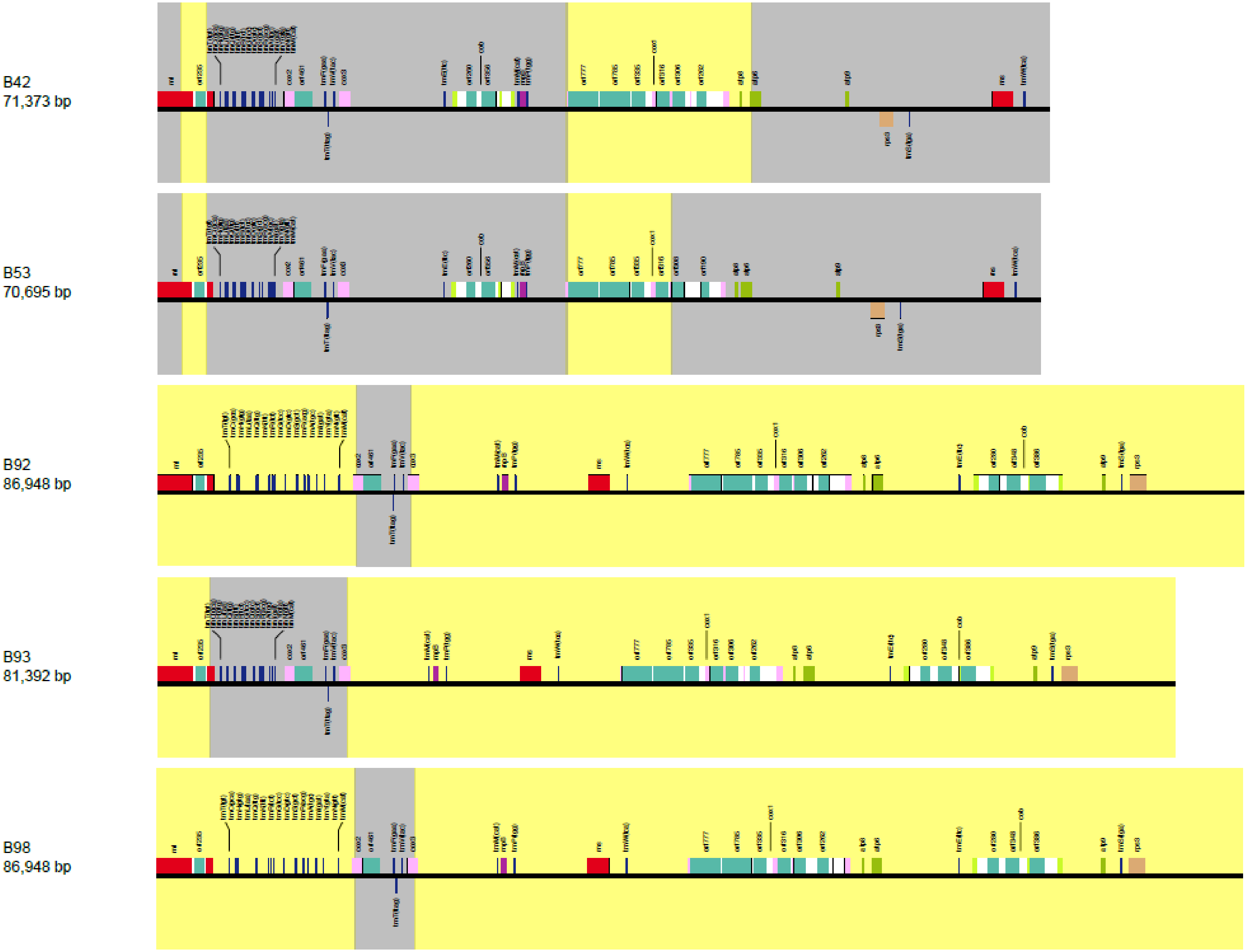

Supplementary Fig. 6S. Linear maps of the recombinant (chimeric) mitogenomes. Yellow: *S. cerevisiae* sequence. Grey: *S. uvarum* sequence

Strain	Gene									
	COB	*COX1*	*COX2*	*COX3*	*ATP6*	*ATP8*	*ATP9*	*rps3/var1*	*rns*	*rn1*
S. c. **11-170**										
S. u. **11-522**										
H1S2					rec					rec
H1S6				rec						rec
H1S15				rec						rec
H1S24		rec								rec
A4		rec								rec
A27		rec								rec
A33		rec								rec
A35		rec								rec
A36							rec			rec
A37					rec					rec
A38				rec						rec
Cu-4.1										rec
Cu-4.8		rec								rec
B2				rec						rec
B4		rec								rec
B11				rec						rec
B16			rec	rec						
B20				rec						
B24		rec								rec
B25										rec
B29		rec								rec
B36				rec						rec
B38			rec	rec						
B42		rec			rec					rec
B53		rec								rec
B92			rec	rec						
B93				rec						rec
B98			rec	rec						
	S.u.			*S.c.*			Recombined (chimeric)		rec	

Supplementary Fig. 7S. Combinations of parental and chimeric genes in recombinant mitotypes.

Atp6

```
S. c. 170    ----MIIMFNLLNTYITSPLDQFEIRTLFGLQSSFIDLSCLNLTTFSLYTIIVLLVITSL
A37          ----MIIMFNLLNTYITSPLDQFEIRTLFGLQSSFIDLSCLNLTTFSLYTIIVLLVILCL
B42          ----MIIMFNLLNTYITSPLDQFEIRTLFGLQSSFIDLSCLNLTTFSLYTIIVLLVILCL
S2           ----MIIMFNLLNTYITSPLDQFEIRTLFGLQSSFIDLSCLNLTTFSLYTIIVLLVILCL
S. u. 522    MVILLKFMFNLLNTYITSPLDQFEIRTLLGLQSSFIDLSCLNLTTFSLYTIIVLLVILCL
                    *********************:**************************** .*

S. c. 170    YTLTNNNNKIIGSRWLISQEAIYDTIMNMTKGQIGGKNWGLYFPMIFTLFMFIFIANLIS
A37          YTLTTNNNKIIGSRWLISQEAIYDTIMNMVKGQIGGKNWGLYFPMIFTFFMFIFVANLIS
B42          YTLTTNNNKIIGSRWLISQEAIYDTIMNMVKGQIGGKNWGLYFPMIFTFFMFIFVANLIS
S2           YTLTTNNNKIIGSRWLISQEAIYDTIMNMVKGQIGGKNWGLYFPMIFTFFMFIFVANLIS
S. u, 522    YTLTTNNNKIIGSRWLISQEAIYDTIMNMVKGQIGGKNWGLYFPMIFTFFMFIFVANLIS
             ****.************************.******************:*****:*****

S. c. 170    MIPYSFALSAHLVFIISLSIVIWLGNTILGLYKHGWVFFSLFVPAGTPLPLVPLLVIIET
A37          MIPYSFALSAHLVFIISLSIIIWLGNTILGLYKHGWTFFSLFVPTGTPLPLVPLLVIIET
B42          MIPYSFALSAHLVFIISLSIIIWLGNTILGLYKHGWTFFSLFVPTGTPLPLVPLLVIIET
S2           MIPYSFALSAHLVFIISLSIIIWLGNTILGLYKHGWTFFSLFVPTGTPLPLVPLLVIIET
S. u. 522    MIPYSFALSAHLVFIISLSIIIWLGNTILGLYKHGWTFFSLFVPTGTPLPLVPLLVIIET
             ********************:***************.*******:***************

S. c. 170    LSYFARAISLGLRLGSNILAGHLLMVILAGLTFNFMLINLFTLVFGFVPLAMILAIMMLE
A37          LSYIARSISLGLRLGSNILAGHLLMIILAGLTFNFMLINLFTLVFGFVPLAMILAIMILE
B42          LSYIARSISLGLRLGSNILAGHLLMIILAGLTFNFMLINLFTLVFGFVPLAMILAIMILE
S2           LSYIARSISLGLRLGSNILAGHLLMIILAGLTFNFMLINLFTLVFGFVPLAMILAIMILE
S. u. 522    LSYIARSISLGLRLGSNILAGHLLMIILAGLTFNFMLINLFTLVFGFVPLAMILAIMILE
             ***:**:******************:*******************************:**

S. c. 170    FAIGIIQGYVWAILTASYLKDAVYLH
A37          FAIGIIQSYVWCILTASYLKDAVYLH
B42          FAIGIIQSYVWCILTASYLKDAVYLH
S2           FAIGIIQSYVWCILTASYLKDAVYLH
S. u. 522    FAIGIIQSYVWCILTASYLKDAVYLH
             *******.***.**************
```

Atp9

```
S. c. 170    MN-IMQLVLAAKYIGAGISTIGLLGAGIGIAIVFAALINGVSRNPSIKDTVFPMAILGFA
A36          MN-IMQLVLAAKYIGAGISTIGLLGAGIGIAIVFAALINGVSRNPSIKDTVFPMAILGFA
S. u. 522    MNKIMQLVLAAKYIGAGISTIGLLGAGIGIAIVFAALINGVSRNPSIKDTVFPMAILGFA
             ** *********************************************************

S. c. 170    LSEATGLFCLMVSFLLLFGV
A36          LSEATGLFCLMVSFLLLFGV
S. u. 522    LSEATGLFCLMVSFLLLFGV
             ********************
```

Cox1

```
S. c. 170    MVQRWLYSTNAKDIAVLYFMLAIFSGMAGTAMSLIIRLELAAPGSQYLHGNSQLFNVLVV
A4           MVQRWLYSTNAKDIAVLYFMLAIFSGMAGTAMSLIIRLELAAPGSQYLHGNSQLFNVLVV
A27          MVQRWLYSTNAKDIAVLYFMLAIFSGMAGTAMSLIIRLELAAPGSQYLHGNSQLFNVLVV
A33          MVQRWLYSTNAKDIAVLYFMLAIFSGMAGTAMSLIIRLELAAPGSQYLHGNSQLFNVLVV
A35          MVQRWLYSTNAKDIAVLYFMLAIFSGMAGTAMSLIIRLELAAPGSQYLQGNSQLFNVLVV
```

```
B4              MVQRWLYSTNAKDIAVLYFMLAIFSGMAGTAMSLIIRLELAAPGSQYLHGNSQLFNVLVV
B24             MVQRWLYSTNAKDIAVLYFMLAIFSGMAGTAMSLIIRLELAAPGSQYLHGNSQLFNVLVV
B29             MVQRWLYSTNAKDIAVLYFMLAIFSGMAGTAMSLIIRLELAAPGSQYLHGNSQLFNVLVV
B42             MVQRWLYSTNAKDIAVLYFMLAIFSGMAGTAMSLIIRLELAAPGSQYLHGNSQLFNVLVV
B53             MVQRWLYSTNAKDIAVLYFMLAIFSGMAGTAMSLIIRLELAAPGSQYLHGNSQLFNVLVV
Cu4.8           MVQRWLYSTNAKDIAVLYFMLAIFSGMAGTAMSLIIRLELAAPGSQYLHGNSQLFNVLVV
S24             MVQRWLYSTNAKDIAVLYFMLAIFSGMAGTAMSLIIRLELAAPGSQYLHGNSQLFNVLVV
S. u. 522       MVQRWLYSTNAKDIAVLYFMLAIFSGMAGTAMSLIIRLELAAPGSQYLQGNAQLFNVLVV
                ************************************************:**:********

S. c. 170       GHAVLMIFFLVMPALIGGFGNYLLPLMIGATDTAFPRINNIAFWVLPMGLVCLVTSTLVE
A4              GHAVLMIFFLVMPALIGGFGNYLLPLMIGATDTAFPRINNIAFWVLPMGLVCLVTSTLVE
A27             GHAVLMIFFLVMPALIGGFGNYLLPLMIGATDTAFPRINNIAFWVLPMGLVCLVTSTLVE
A33             GHAVLMIFFLVMPALIGGFGNYLLPLMIGATDTAFPRINNIAFWVLPMGLVCLVTSTLVE
A35             GHAVLMIFFLVMPALIGGFGNYLLPLMIGATDTAFPRINNIAFWVLPMGLVCLVTSTLVE
B4              GHAVLMIFFLVMPALIGGFGNYLLPLMIGATDTAFPRINNIAFWVLPMGLVCLVTSTLVE
B24             GHAVLMIFFLVMPALIGGFGNYLLPLMIGATDTAFPRINNIAFWVLPMGLVCLVTSTLVE
B29             GHAVLMIFFLVMPALIGGFGNYLLPLMIGATDTAFPRINNIAFWVLPMGLVCLVTSTLVE
B42             GHAVLMIFFLVMPALIGGFGNYLLPLMIGATDTAFPRINNIAFWVLPMGLVCLVTSTLVE
B53             GHAVLMIFFLVMPALIGGFGNYLLPLMIGATDTAFPRINNIAFWVLPMGLVCLVTSTLVE
Cu4.8           GHAVLMIFFLVMPALIGGFGNYLLPLMIGATDTAFPRINNIAFWVLPMGLVCLVTSTLVE
S24             GHAVLMIFFLVMPALIGGFGNYLLPLMIGATDTAFPRINNIAFWVLPMGLVCLVTSTLVE
S. u. 522       GHAVLMIFFLVMPSLIGGFGNYLLPLMIGATDTAFPRINNIAFWVLPMGLVCLVTSTLVE
                *************:**********************************************

S. c. 170       SGAGTGWTVYPPLSSIQAHSGPSVDLAIFALHLTSISSLLGAINFIVTTLNMRTNGMTMH
A4              SGAGTGWTVYPPLSSIQAHSGPSVDLAIFALHLTSISSLLGAINFIVTTLNMRTNGMTMH
A27             SGAGTGWTVYPPLSSIQAHSGPSVDLAIFALHLTSISSLLGAINFIVTTLNMRTNGMTMH
A33             SGAGTGWTVYPPLSSIQAHSGPSVDLAIFALHLTSISSLLGAINFIVTTLNMRTNGMTMH
A35             SGAGTGWTVYPPLSSIQAHSGPSVDLAIFALHLTSISSLLGAINFIVTTLNMRTNGMTMH
B4              SGAGTGWTVYPPLSSIQAHSGPSVDLAIFALHLTSISSLLGAINFIVTTLNMRTNGMTMH
B24             SGAGTGWTVYPPLSSIQAHSGPSVDLAIFALHLTSISSLLGAINFIVTTLNMRTNGMTMH
B29             SGAGTGWTVYPPLSSIQAHSGPSVDLAIFALHLTSISSLLGAINFIVTTLNMRTNGMTMH
B42             SGAGTGWTVYPPLSSIQAHSGPSVDLAIFALHLTSISSLLGAINFIVTTLNMRTNGMTMH
B53             SGAGTGWTVYPPLSSIQAHSGPSVDLAIFALHLTSISSLLGAINFIVTTLNMRTNGMTMH
Cu4.8           SGAGTGWTVYPPLSSIQAHSGPSVDLAIFALHLTSISSLLGAINFIVTTLNMRTNGMTMH
S24             SGAGTGWTVYPPLSSIQAHSGPSVDLAIFALHLTSISSLLGAINFIVTTLNMRTNGMTMH
S. u. 522       SGAGTGWTVYPPLSSIQAHSGPSVDLAIFALHLTSISSLLGAINFIVTTLNMRTNGMTMH
                ************************************************************

S. c. 170       KLPLFVWSIFITAFLLLLSLPVLSAGITMLLLDRNFNTSFFEVAGGGDPILYEHLFWFFG
A4              KLPLFVWSIFITAFLLLLSLPVLSAGITMLLLDRNFNTSFFEVAGGGDPILYEHLFWFFG
A27             KLPLFVWSIFITAFLLLLSLPVLSAGITMLLLDRNFNTSFFEVAGGGDPILYEHLFWFFG
A33             KLPLFVWSIFITAFLLLLSLPVLSAGITMLLLDRNFNTSFFEVAGGGDPILYEHLFWFFG
A35             KLPLFVWSIFITAFLLLLSLPVLSAGITMLLLDRNFNTSFFEVAGGGDPILYEHLFWFFG
B4              KLPLFVWSIFITAFLLLLSLPVLSAGITMLLLDRNFNTSFFEVAGGGDPILYEHLFWFFG
B24             KLPLFVWSIFITAFLLLLSLPVLSAGITMLLLDRNFNTSFFEVAGGGDPILYEHLFWFFG
B29             KLPLFVWSIFITAFLLLLSLPVLSAGITMLLLDRNFNTSFFEVAGGGDPILYEHLFWFFG
B42             KLPLFVWSIFITAFLLLLSLPVLSAGITMLLLDRNFNTSFFEVAGGGDPILYEHLFWFFG
B53             KLPLFVWSIFITAFLLLLSLPVLSAGITMLLLDRNFNTSFFEVAGGGDPILYEHLFWFFG
Cu4.8           KLPLFVWSIFITAFLLLLSLPVLSAGITMLLLDRNFNTSFFEVAGGGDPILYEHLFWFFG
S24             KLPLFVWSIFITAFLLLLSLPVLSAGITMLLLDRNFNTSFFEVAGGGDPILYEHLFWFFG
S. u. 522       KLPLFVWSIFITAFLLLLSLPVLSAGITMLLLDRNFNTSFFEVAGGGDPILYEHLFWFFG
                ************************************************************

S. c. 170       HPEVYILIIPGFGIISHVVSTYSKKPVFGEISMVYAMASIGLLGFLVWSHHMYIVGLDAD
A4              HPEVYILIIPGFGIISHVVSTYSKKPVFGEISMVYAMASIGLLGFLVWSHHMYIVGLDAD
A27             HPEVYILIIPGFGIISHVVSTYSKKPVFGEISMVYAMASIGLLGFLVWSHHMYIVGLDAD
A33             HPEVYILIIPGFGIISHVVSTYSKKPVFGEISMVYAMASIGLLGFLVWSHHMYIVGLDAD
A35             HPEVYILIIPGFGIISHVVSTYSKKPVFGEISMVYAMASIGLLGFLVWSHHMYIVGLDAD
B4              HPEVYILIIPGFGIISHVVSTYSKKPVFGEISMVYAMASIGLLGFLVWSHHMYIVGLDAD
```

```
B24             HPEVYILIIPGFGIISHVVSTYSKKPVFGEISMVYAMASIGLLGFLVWSHHMYIVGLDAD
B29             HPEVYILIIPGFGIISHVVSTYSKKPVFGEISMVYAMASIGLLGFLVWSHHMYIVGLDAD
B42             HPEVYILIIPGFGIISHVVSTYSKKPVFGEISMVYAMASIGLLGFLVWSHHMYIVGLDAD
B53             HPEVYILIIPGFGIISHVVSTYSKKPVFGEISMVYAMASIGLLGFLVWSHHMYIVGLDAD
Cu4.8           HPEVYILIIPGFGIISHVVSTYSKKPVFGEISMVYAMASIGLLGFLVWSHHMYIVGLDAD
S24             HPEVYILIIPGFGIISHVVSTYSKKPVFGEISMVYAMASIGLLGFLVWSHHMYIVGLDAD
S. u. 522       HPEVYILIIPGFGIISHVVSTYSKKPVFGEISMVYAMASIGLLGFLVWSHHMYIVGLDAD
                ************************************************************

S. c. 170       TRAYFTSATMIIAIPTGIKIFSWLATIHGGSIRLATPMLYAIAFLFLFTMGGLTGVALAN
A4              TRAYFTSATMIIAIPTGIKIFSWLATIYGGSIRLATPMLYAIAFLFLFTMGGLTGVALAN
A27             TRAYFTSATMIIAIPTGIKIFSWLATIYGGSIRLATPMLYAIAFLFLFTMGGLTGVALAN
A33             TRAYFTSATMIIAIPTGIKIFSWLATIHGGSIRLATPMLYAIAFLFLFTMGGLTGVALAN
A35             TRAYFTSATMIIAIPTGIKIFSWLATIYGGSIRLATPMLYAIAFLFLFTMGGLTGVALAN
B4              TRAYFTSATMIIAIPTGIKIFSWLATIHGGSIRLATPMLYAIAFLFLFTMGGLTGVALAN
B24             TRAYFTSATMIIAIPTGIKIFSWLATIYGGSIRLATPMLYAIAFLFLFTMGGLTGVALAN
B29             TRAYFTSATMIIAIPTGIKIFSWLATIYGGSIRLATPMLYAIAFLFLFTMGGLTGVALAN
B42             TRAYFTSATMIIAIPTGIKIFSWLATIHGGSIRLATPMLYAIAFLFLFTMGGLTGVALAN
B53             TRAYFTSATMIIAIPTGIKIFSWLATIYGGSIRLATPMLYAIAFLFLFTMGGLTGVALAN
Cu4.8           TRAYFTSATMIIAIPTGIKIFSWLATIYGGSIRLATPMLYAIAFLFLFTMGGLTGVALAN
S24             TRAYFTSATMIIAIPTGIKIFSWLATIYGGSIRLATPMLYAIAFLFLFTMGGLTGVALAN
S. u. 522       TRAYFTSATMIIAIPTGIKIFSWLATIYGGSIRLATPMLYAIAFLFLFTMGGLTGVALAN
                ***************************:********************************

S. c. 170       ASLDVAFHDTYYVVGHFHYVLSMGAIFSLFAGYYYWSPQILGLNYNEKLAQIQFWLIFIG
A4              ASLDVAFHDTYYVVGHFHYVLSMGAIFSLFAGYYYWSPQILGLNYNEKLAQIQFWLIFIG
A27             ASLDVAFHDTYYVVGHFHYVLSMGAIFSLFAGYYYWSPQILGLNYNEKLAQIQFWLIFIG
A33             ASLDVAFHDTYYVVGHFHYVLSMGAIFSLFAGYYYWSPQILGLNYNEKLAQIQFWLIFIG
A35             ASLDVAFHDTYYVVGHFHYVLSMGAIFSLFAGYYYWSPQILGLNYNEKLAQIQFWLIFIG
B4              ASLDVAFHDTYYVVGHFHYVLSMGAIFSLFAGYYYWSPQILGLNYNEKLAQIQFWLIFIG
B24             ASLDVAFHDTYYVVGHFHYVLSMGAIFSLFAGYYYWSPQILGLNYNEKLAQIQFWLIFIG
B29             ASLDVAFHDTYYVVGHFHYVLSMGAIFSLFAGYYYWSPQILGLNYNEKLAQIQFWLIFIG
B42             ASLDVAFHDTYYVVGHFHYVLSMGAIFSLFAGYYYWSPQILGLNYNEKLAQIQFWLIFIG
B53             ASLDVAFHDTYYVVGHFHYVLSMGAIFSLFAGYYYWSPQILGLNYNEKLAQIQFWLIFIG
Cu4.8           ASLDVAFHDTYYVVGHFHYVLSMGAIFSLFAGYYYWSPQILGLNYNEKLAQIQFWLIFIG
S24             ASLDVAFHDTYYVVGHFHYVLSMGAIFSLFAGYYYWSPQILGLNYNEKLAQIQFWLIFIG
S. u. 522       ASLDVAFHDTYYVVGHFHYVLSMGAIFSLFAGYYYWSPQILGLNYNEKLAQIQFWLIFIG
                ************************************************************

S. c. 170       ANVIFFPMHFLGINGMPRRIPDYPDAFAGWNYVASIGSFIATLSLFLFIYILYDQLVNGL
A4              ANVIFFPMHFLGINGMPRRIPDYPDAFAGWNYVASIGSFIATISLFLFIYILYDQLVNGL
A27             ANVIFFPMHFLGINGMPRRIPDYPDAFAGWNYVASIGSFIATISLFLFIYILYDQLVNGL
A33             ANVIFFPMHFLGINGMPRRIPDYPDAFAGWNYVASIGSFIATISLFLFIYILYDQLVNGL
A35             ANVIFFPMHFLGINGMPRRIPDYPDAFAGWNYVASIGSFIATISLFLFIYILYDQLVNGL
B4              ANVIFFPMHFLGINGMPRRIPDYPDAFAGWNYVASIGSFIATISLFLFIYILYDQLVNGL
B24             ANVIFFPMHFLGINGMPRRIPDYPDAFAGWNYVASIGSFIATISLFLFIYILYDQLVNGL
B29             ANVIFFPMHFLGINGMPRRIPDYPDAFAGWNYVASIGSFIATISLFLFIYILYDQLVNGL
B42             ANVIFFPMHFLGINGMPRRIPDYPDAFAGWNYVASIGSFIATLSLFLFIYILYDQLVNGL
B53             ANVIFFPMHFLGINGMPRRIPDYPDAFAGWNYVASIGSFIATISLFLFIYILYDQLVNGL
Cu4.8           ANVIFFPMHFLGINGMPRRIPDYPDAFAGWNYVASIGSFIATISLFLFIYILYDQLVNGL
S24             ANVIFFPMHFLGINGMPRRIPDYPDAFAGWNYVASIGSFIATISLFLFIYILYDQLVNGL
S. u. 522       ANVIFFPMHFLGINGMPRRIPDYPDAFAGWNYVASIGSFIATISLFLFIYILYDQLVNGL
                ******************************************:*****************

S. c. 170       NNKVNNKSVIYNKAPDFVESNTIFNLNTVKSSSIEFLLTSPPAVHSFNTPAVQS
A4              NNKVTNKSVVYSKAPDFVESNMIFNLNTVKSSSIEFLLTSPPAVHSFNTPAVQS
A27             NNKVTNKSVVYSKAPDFVESNMIFNLNTVKSSSIEFLLTSPPAVHSFNTPAVQS
A33             NNKVTNKSVVYSKAPDFVESNMIFNLNTVKSSSIEFLLTSPPAVHSFNTPAVQS
A35             NNKVTNKSVVYSKAPDFVESNMIFNLNTVKSSSIEFLLTSPPAVHSFNTPAVQS
B4              NNKVTNKSVVYSKAPDFVESNMIFNLNTVKSSSIEFLLTSPPAVHSFNTPAVQS
B24             NNKVTNKSVVYSKAPDFVESNMIFNLNTVKSSSIEFLLTSPPAVHSFNTPAVQS
```

```
B29             NNKVTNKSVVYSKAPDFVESNMIFNLNTVKSSSIEFLLTSPPAVHSFNTPAVQS
B42             NNKVNNKSVIYNKAPDFVESNTIFNLNTVKSSSIEFLLTSPPAVHSFNTPAVQS
B53             NNKVTNKSVVYSKAPDFVESNMIFNLNTVKSSSIEFLLTSPPAVHSFNTPAVQS
Cu4.8           NNKVTNKSVVYSKAPDFVESNMIFNLNTVKSSSIEFLLTSPPAVHSFNTPAVQS
S24             NNKVTNKSVVYSKAPDFVESNMIFNLNTVKSSSIEFLLTSPPAVHSFNTPAVQS
S. u. 522       NNKVTNKSVVYSKAPDFVESNMIFNLNTVKSSSIEFLLTSPPAVHSFNTPAVQS
                ****.****:*.********* ********************************
```

Cox2

```
S. c. 170       MLDLLRLQLTTFIMNDVPTPYACYFQDSATPNQEGILELHDNIMFYLLVILGLVSWMLYT
B16             MLDLLRLQLTTFIMNDVPTPYACYFQDSATPNQEGILELHDNIMFYLLVILGLVSWMLYT
B38             MLDLLRLQLTTFIMNDVPTPYACYFQDSATPNQEGILELHDNIMFYLLVILGLVSWMLYT
B92             MLDLLRLQLTTFIMNDVPTPYACYFQDSATPNQEGILELHDNIMFYLLVILGLVSWMLYT
B98             MLDLLRLQLTTFIMNDVPTPYACYFQDSATPNQEGILELHDNIMFYLFVILGLVSWMLYT
S. u. 522       MLDLLRLQLTTVIMNDVPTPYACYFQDSATPNQEGILELHDNIMFYLFVILGLVSWMLYT
                ***********.***********************************:************

S. c. 170       IVMTYSKNPIAYKYIKHGQTIEVIWTIFPAVILLIIAFPSFILLYLCDEVISPAMTIKAI
B16             IVRTYSNNPIAYKYIKHGQTIEVIWTIFPAVVLLIIAFPSFILLYLCDEVISPAMTIKAI
B38             IVMTYSKNPIAYKYIKHGQTIEVIWTIFPAVILLIIAFPSFILLYLCDEVISPAMTIKAI
B92             IVRTYSNNPIAYKYIKHGQTIEVIWTIFPAVVLLIIAFPSFILLYLCDEVISPAMTIKAI
B98             IVRTYSNNPIAYKYIKHGQTIEVIWTIFPAVVLLIIAFPSFILLYLCDEVISPAMTIKAI
S. u. 522       IVRTYSNNPIAYKYIKHGQTIEVIWTIFPAVVLLIIAFPSFILLYLCDEVISPAMTIKAI
                ** ***:************************:****************************

S. c. 170       GYQWYWKYEYSDFINDSGETVEFESYVIPDELLEEGQLRLLDTDTSMVVPVDTHIRFVVT
B16             GYQWYWKYEYSDFINDSGETVEFESYVIPDDLLEEGQLRLLDTDTSIVVPVDTHIRFVVT
B38             GYQWYWKYEYSDFINDSGETVEFESYVIPDDLLEEGQLRLLDTDTSIVVPVDTHIRFVVT
B92             GYQWYWKYEYSDFINDSGETVEFESYVIPDDLLEEGQLRLLDTDTSIVVPVDTHIRFVVT
B98             GYQWYWKYEYSDFINDSGETVEFESYVIPDDLLEEGQLRLLDTDTSIVVPVDTHIRFVVT
S. u. 522       GYQWYWKYEYSDFINDSGETVEFESYVIPDDLLEEGQLRLLDTDTSIVVPVDTHIRFVVT
                ******************************:***************:*************

S. c. 170       AADVIHDFAIPSLGIKVDATPGRLNQVSALIQREGVFYGACSELCGTGHANMPIKIEAVS
B16             AADVIHDFAIPSLGIKVDATPGRLNQVSALIQREGVFYGNCSELCGTGHANMPIKIEAVS
B38             AADVIHDFAIPSLGIKVDATPGRLNQVSALIQREGVFYGNCSELCGTGHANMPIKIEAVS
B92             AADVIHDFAIPSLGIKVDATPGRLNQVSALIQREGVFYGNCSELCGTGHANMPIKIEAVS
B98             AADVIHDFAIPSLGIKVDATPGRLNQVSALIQREGVFYGNCSELCGTGHANMPIKIEAVS
S. u. 522       AADVIHDFAIPSLGIKVDATPGRLNQVSALIQREGVFYGNCSELCGTGHANMPIKIEAVS
                *************************************** ********************

S. c. 170       LPKFLEWLNEQ
B16             LPKFLEWLNEQ
B38             LPKFLEWLNEQ
B92             LPKFLEWLNEQ
B98             LPKFLEWLNEQ
S. u. 522       LPKFLEWLNEQ
                ***********
```

Cox3

```
S. c. 170       MTHLERSRHQQHPFHMVMPSPWPIVVSFALLSLALSTALTMHGYIGNMNMVYLALFVLLT
A38             MTHLERSRHQQHPFHMVMPSPWPIVVSFALLSLALSTALTMHGYIGNMNMVYLALFVLLT
B2              MTHLERSRHQQHPFHMVMPSPWPIVVSFALLSLALSTALTMHGYIGNMNMVYLALFVLLT
B11             MTHLERSRHQQHPFHMVMPSPWPIVVSFALLSLALSTALTMHGYIGNMNMVYLALFVLLT
B16             MTHLERSRHQQHPFHMVMPSPWPIVVSFALLSLALSTALTMHGYIGNMNMVYLALFVLLT
B20             MTHLERSRHQQHPFHMVMPSPWPIVVSFALLSLALSTALTMHGYIGNMNMVYLALFVLLT
B36             MTHLERSRHQQHPFHMVMPSPWPIVVSFALLSLALSTALTMHGYIGNMNMVYLALFVLLT
B38             MTHLERSRHQQHPFHMVMPSPWPIVVSFALLSLALSTALTMHGYIGNMNMVYLALFVLLT
```

```
B92                 MTHLERSRHQQHPFHMVMPSPWPIVVSFALLSLALSTALTMHGYIGNMNMVYLALFVLLT
B93                 MTHLERSRHQQHPFHMVMPSPWPIVVSFALLSLALSTALTMHGYIGNMNMVYLALFVLLT
B98                 MTHLERSRHQQHPFHMVMPSPWPIVVSFALLSLALSTALTMHGYIGNMNMVYLALFVLLT
S6                  MTHLERSRHQQHPFHMVMPSPWPIVVSFALLSLALSTALTMHGYIGNMNMVYLALFVLLT
S15                 MTHLERSRHQQHPFHMVMPSPWPIVVSFALLSLALSTALTMHGYIGNMNMVYLALFVLLT
S. u. 522           MTHLERSRHQQHPFHMVMPSPWPIVVSFALLSLALSTALTMHGYIGNMNMVYLALFVLLT
                    ************************************************************

S. c. 170           SSILWFRDIVAEATYLGDHTMAVRKGINLGFLMFVLSEVLIFAGLFWAYFHSAMSPDVTL
A38                 SSILWFRDIVAEATYLGDHTMAVRKGINLGFLMFVLSEVLIFAGLFWAYFHSAMSPDVTL
B2                  SSILWFRDIVSEATYLGEHTIAVRKGINLGFLMFVLSEVLIFAGLFWAYFHSAMSPDVTL
B11                 SSILWFRDIVSEATYLGEHTIAVRKGINLGFLMFVLSEVLIFAGLFWAYFHSAMSPDVTL
B16                 SSILWFRDIVSEATYLGEHTIAVRKGINLGFLMFVLSEVLIFAGLFWAYFHSAMSPDVTL
B20                 SSILWFRDIVSEATYLGEHTIAVRKGINLGFLMFVLSEVLIFAGLFWAYFHSAMSPDVTL
B36                 SSILWFRDIVSEATYLGEHTIAVRKGINLGFLMFVLSEVLIFAGLFWAYFHSAMSPDVTL
B38                 SSILWFRDIVSEATYLGEHTIAVRKGINLGFLMFVLSEVLIFAGLFWAYFHSAMSPDVTL
B92                 SSILWFRDIVAEATYLGDHTMAVRKGINLGFLMFVLSEVLIFAGLFWAYFHSAMSPDVTL
B93                 SSILWFRDIVSEATYLGEHTIAVRKGINLGFLMFVLSEVLIFAGLFWAYFHSAMSPDVTL
B98                 SSILWFRDIVSEATYLGEHTIAVRKGINLGFLMFVLSEVLIFAGLFWAYFHSAMSPDVTL
S6                  SSILWFRDIVSEATYLGEHTIAVRKGINLGFLMFVLSEVLIFAGLFWAYFHSAMSPDVTL
S15                 SSILWFRDIVAEATYLGDHTMAVRKGINLGFLMFVLSEVLIFAGLFWAYFHSAMSPDVTL
S. u. 522           SSILWFRDIVSEATYLGEHTIAVRKGINLGFLMFVLSEVLIFAGLFWAYFHSAMSPDVTL
                    **********:******:**:***************************************

S. c. 170           GACWPPVGIEAVQPTELPLLNTIILLSSGATVTYSHHALIAGNRNKALSGLLITFWLIVI
A38                 GACWPPVGIEAVQPTELPLLNTIILLSSGATVTYSHHALIAGNRNKALSGLLITFWLIVI
B2                  GSCWPPVGIEAVQPTELPLLNTIILLSSGATVTYSHHALIAGNRNKALSGLLITFWLIVI
B11                 GSCWPPVGIEAVQPTELPLLNTIILLSSGATVTYSHHALIAGNRNKALSGLLITFWLIVI
B16                 GSCWPPVGIEAVQPTELPLLNTIILLSSGATVTYSHHALIAGNRNKALSGLLITFWLIVI
B20                 GSCWPPVGIEAVQPTELPLLNTIILLSSGATVTYSHHALIAGNRNKALSGLLITFWLIVI
B36                 GACWPPVGIEAVQPTELPLLNTIILLSSGATVTYSHHALIAGNRNKALSGLLITFWLIVI
B38                 GSCWPPVGIEAVQPTELPLLNTIILLSSGATVTYSHHALIAGNRNKALSGLLITFWLIVI
B92                 GACWPPVGIEAVQPTELPLLNTIILLSSGATVTYSHHALIAGNRNKALSGLLITFWLIVI
B93                 GSCWPPVGIEAVQPTELPLLNTIILLSSGATVTYSHHALIAGNRNKALSGLLITFWLIVI
B98                 GSCWPPVGIEAVQPTELPLLNTIILLSSGATVTYSHHALIAGNRNKALSGLLITFWLIVI
S6                  GSCWPPVGIEAVQPTELPLLNTIILLSSGATVTYSHHALIAGNRNKALSGLLITFWLIVI
S15                 GACWPPVGIEAVQPTELPLLNTIILLSSGATVTYSHHALIAGNRNKALSGLLITFWLIVI
S. u. 522           GSCWPPVGIEAVQPTELPLLNTIILLSSGATVTYSHHALIAGNRNKALSGLLITFWLIVI
                    *:**********************************************************

S. c. 170           FVTCQYIEYTNAAFTISDGVYGSVFYAGTGLHFLHMVMLAAMLGVNYWRMRNYHLTAGHH
A38                 FVTCQYIEYTNAAFTISDGVYGSVFYAGTGLHFLHMVMLAAMLGVNYWRMRNYHLTAGHH
B2                  FVSCQYIEYTNAAFTISDGVYGSVFYAGTGLHFLHMVMLAAMLGVNYWRMRNYHLTAGHH
B11                 FVTCQYIEYTNAAFTISDGVYGSVFYAGTGLHFLHMVMLAAMLGVNYWRMRNYHLTAGHH
B16                 FVSCQYIEYTNAAFTISDGVYGSVFYAGTGLHFLHMVMLAAMLGVNYWRLRNYHLTAGHH
B20                 FVTCQYIEYTNAAFTISDGVYGSVFYAGTGLHFLHMVMLAAMLGVNYWRMRNYHLTAGHH
B36                 FVTCQYIEYTNAAFTISDGVYGSVFYAGTGLHFLHMVMLAAMLGVNYWRMRNYHLTAGHH
B38                 FVSCQYIEYTNAAFTISDGVYGSVFYAGTGLHFLHMVMLAAMLGVNYWRLRNYHLTAGHH
B92                 FVTCQYIEYTNAAFTISDGVYGSVFYAGTGLHFLHMVMLAAMLGVNYWRMRNYHLTAGHH
B93                 FVSCQYIEYTNAAFTISDGVYGSVFYAGTGLHFLHMVMLAAMLGVNYWRLRNYHLTAGHH
B98                 FVTCQYIEYTNAAFTISDGVYGSVFYAGTGLHFLHMVMLAAMLGVNYWRMRNYHLTAGHH
S6                  FVTCQYIEYTNAAFTISDGVYGSVFYAGTGLHFLHMVMLAAMLGVNYWRMRNYHLTAGHH
S15                 FVTCQYIEYTNAAFTISDGVYGSVFYAGTGLHFLHMVMLAAMLGVNYWRMRNYHLTAGHH
S. u. 522           FVSCQYIEYTNAAFTISDGVYGSVFYAGTGLHFLHMVMLAAMLGVNYWRLRNYHLTAGHH
                    **:**********************************************:**********

S. c. 170           VGYETTIIYTHVLDVIWLFLYVTFYWWGV
A38                 VGYETTIIYTHVLDVIWLFLYVTFYWWGV
B2                  VGYETTIIYTHVLDVIWLFLYVTFYWWGV
B11                 VGYETTIIYTHVLDVIWLFLYVTFYWWGV
B16                 VGYETTIIYTHVLDVIWLFLYVTFYWWGV
```

```
B20             VGYETTIIYTHVLDVIWLFLYVTFYWWGV
B36             VGYETTIIYTHVLDVIWLFLYVTFYWWGV
B38             VGYETTIIYTHVLDVIWLFLYVTFYWWGV
B92             VGYETTIIYTHVLDVIWLFLYVTFYWWGV
B93             VGYETTIIYTHVLDVIWLFLYVTFYWWGV
B98             VGYETTIIYTHVLDVIWLFLYVTFYWWGV
S6              VGYETTIIYTHVLDVIWLFLYVTFYWWGV
S15             VGYETTIIYTHVLDVIWLFLYVTFYWWGV
S. u. 522       VGYETTIIYTHVLDVIWLFLYVTFYWWGV
                *****************************
```

Supplementary Fig. 8S. Amino acid sequence alignments of mitochondrial proteins derived from chimeric genes. Amino acids that differ from those of *S. cerevisiae* 10-170 are highlighted in blue.

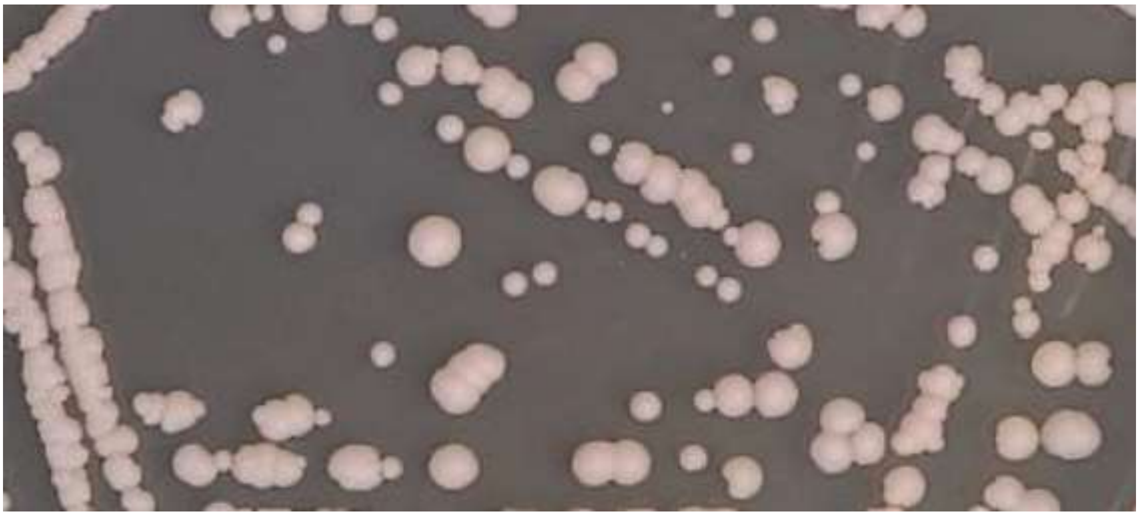

Supplementary Fig. 9S. Large and small colonies in the segregating culture of hybrid B2.

(A)

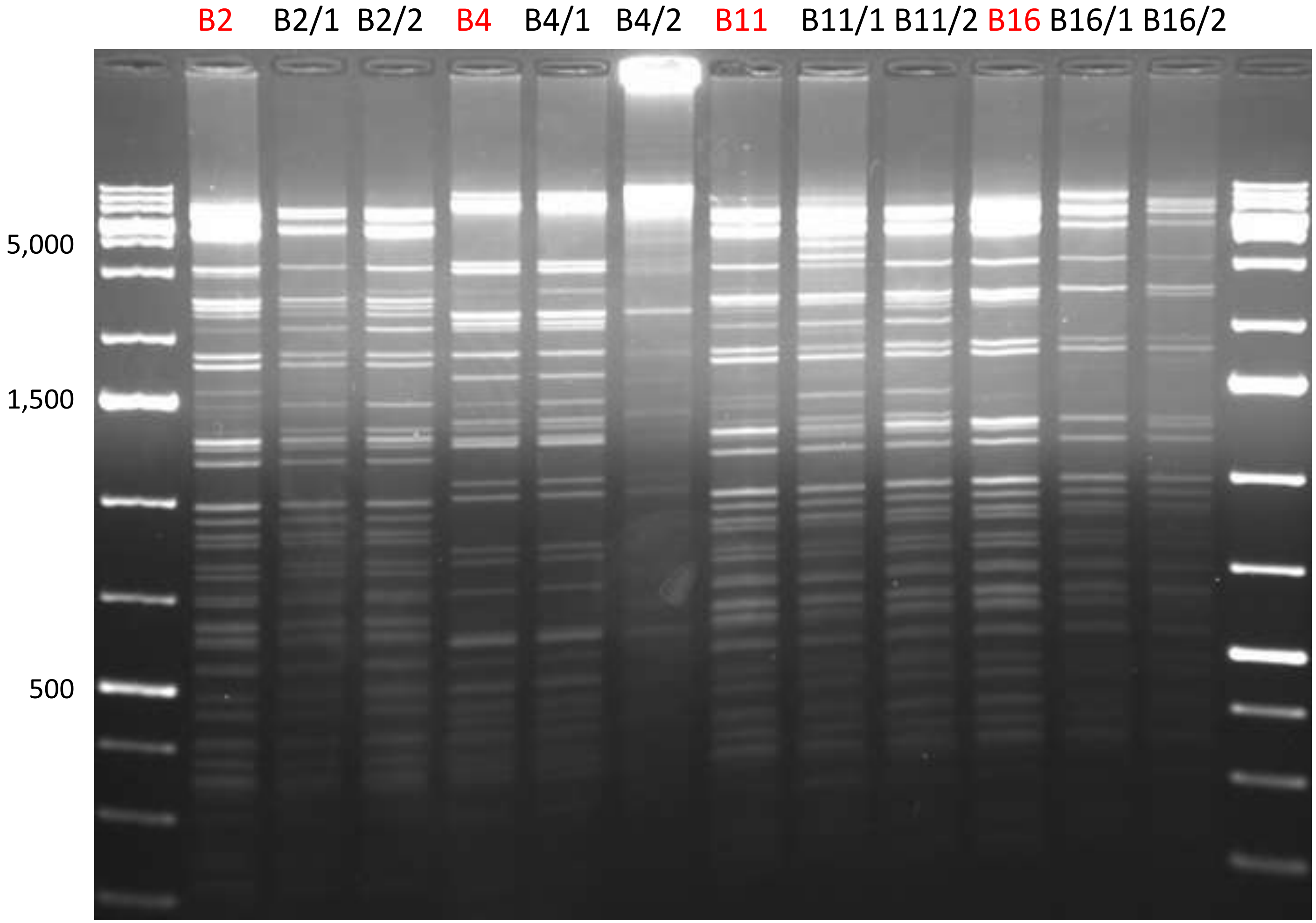

(B)

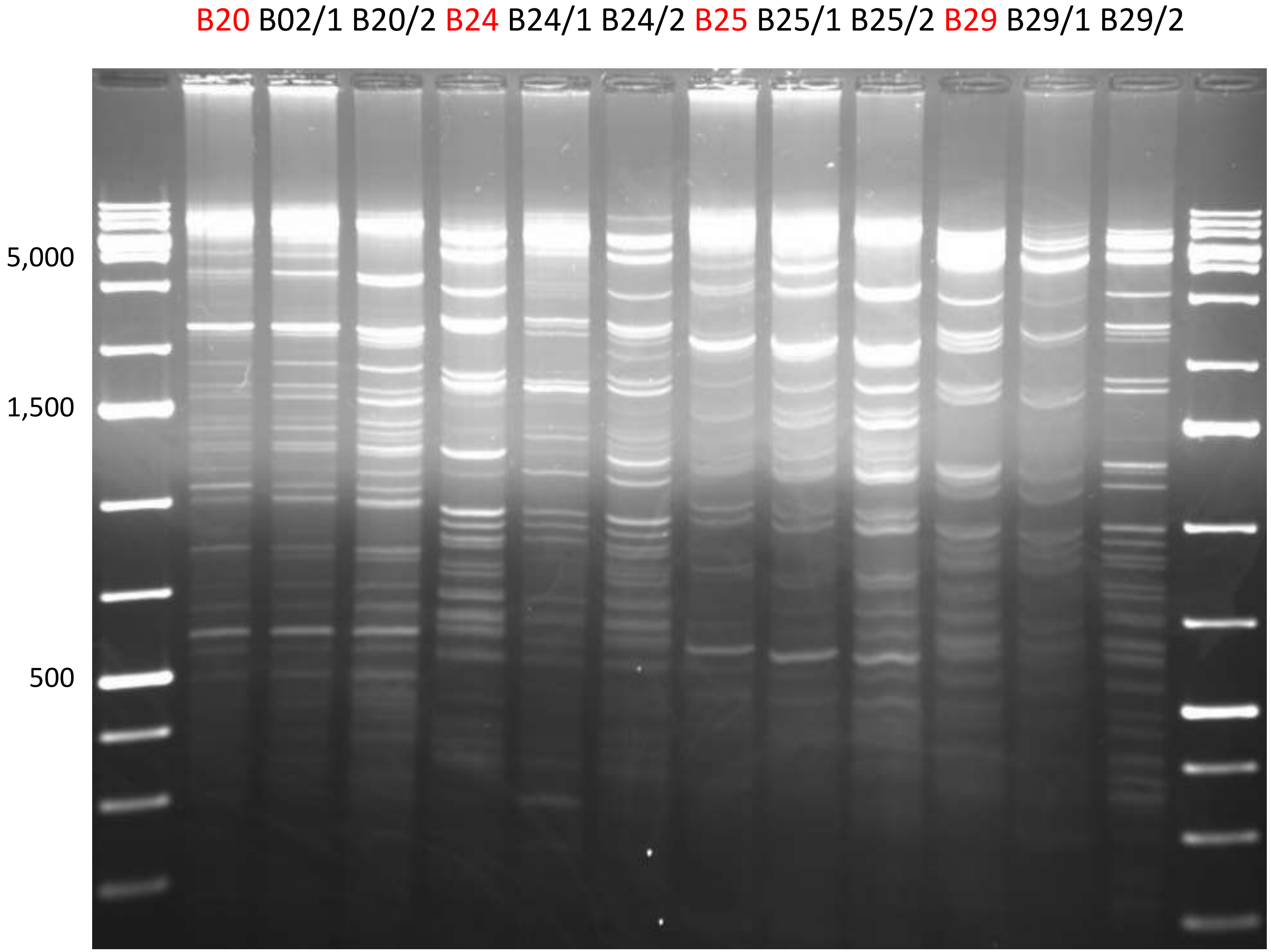
B20 B02/1 B20/2 B24 B24/1 B24/2 B25 B25/1 B25/2 B29 B29/1 B29/2
5,000
1,500
500

(C)

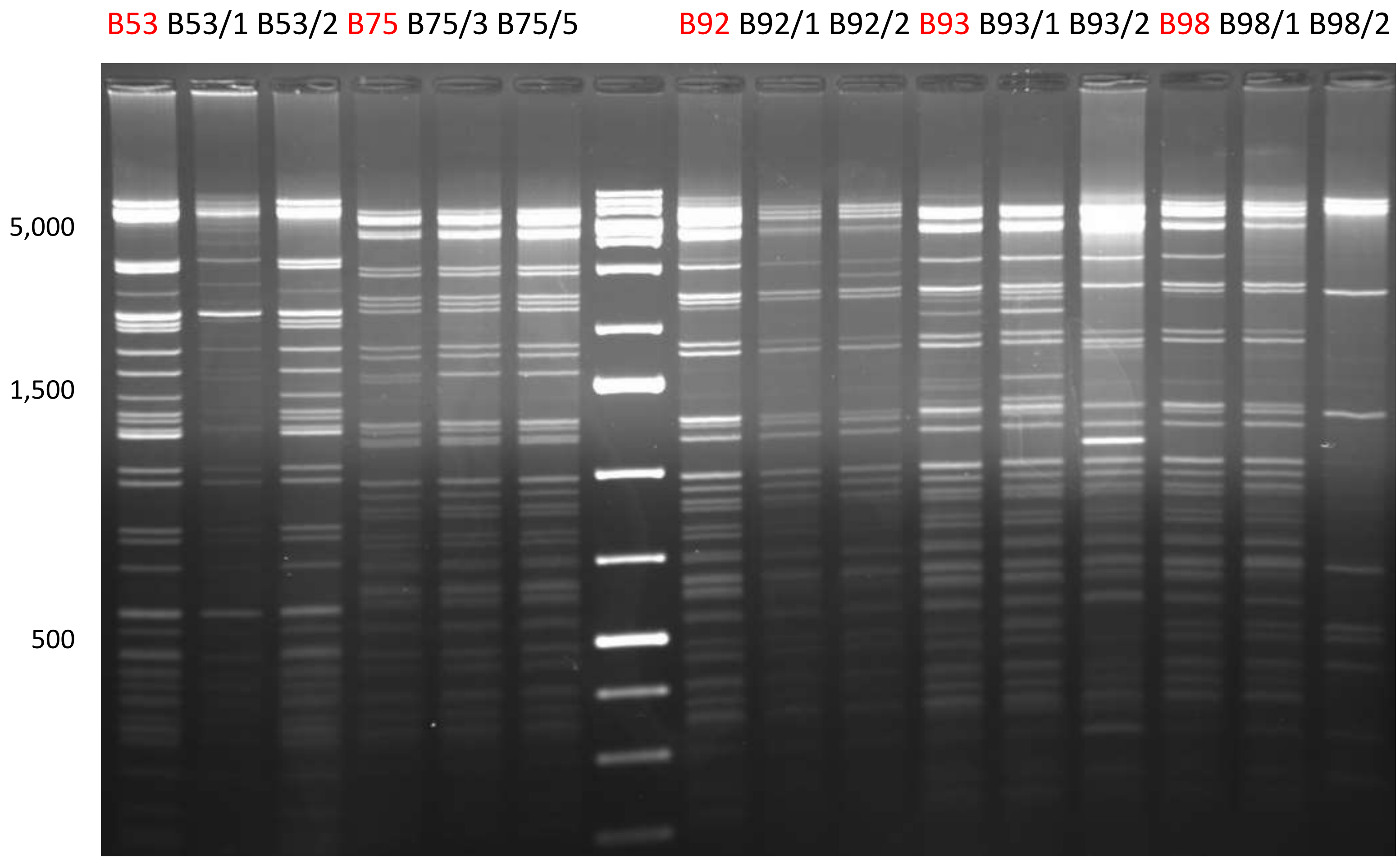

Supplementary Fig. 10S. RFLP patterns of the mitogenomes of hybrids (red) and segregants
