# Supplementary material for: Nucleo-cytoplasmic compatibility in interspecies Saccharomyces hybrids and the destabilisation of the mitogenome by allospecific recombination
Source: Sci Rep. 2026 May 9;16:21279. doi: 10.1038/s41598-026-51924-x (PMC13346842; doi:10.1038/s41598-026-51924-x)

**Non-cropped gel photographs**

**Supplementary Fig. 1S**


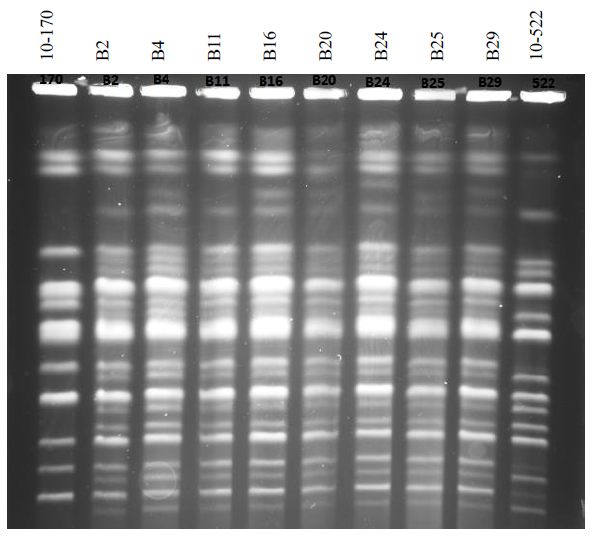


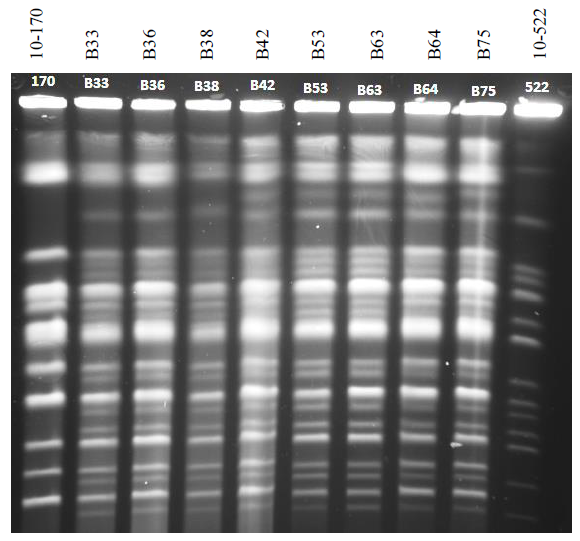


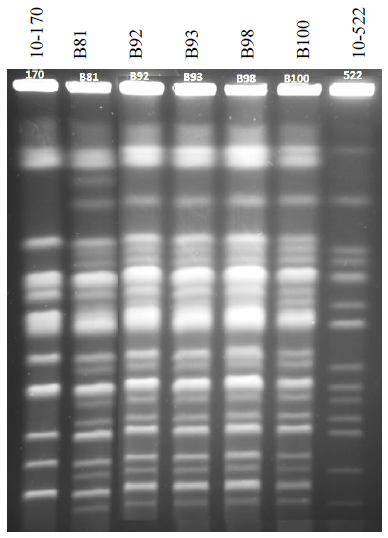


**Supplementary Fig. 2S**


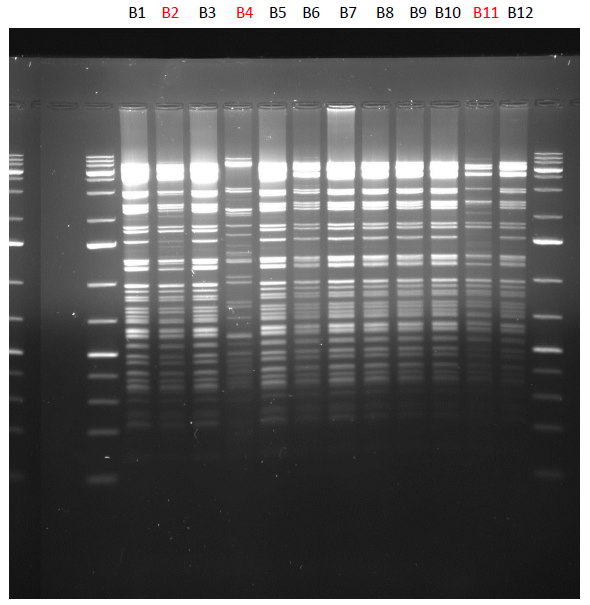


**Supplementary Fig. 3S**


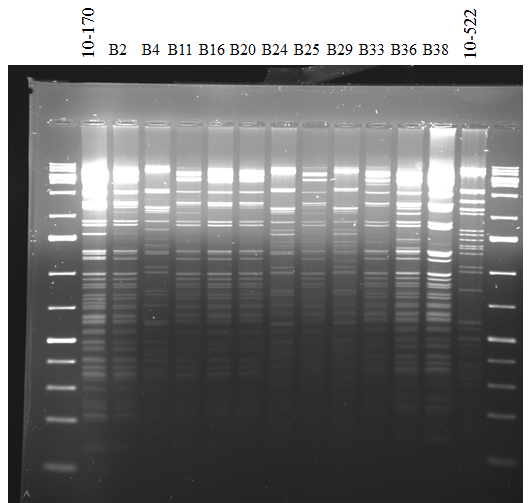


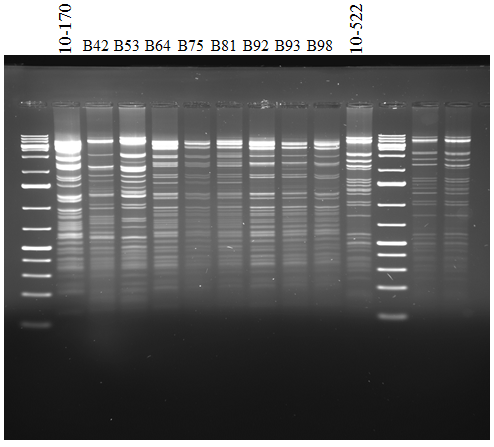


**Supplementary Fig. 10S**


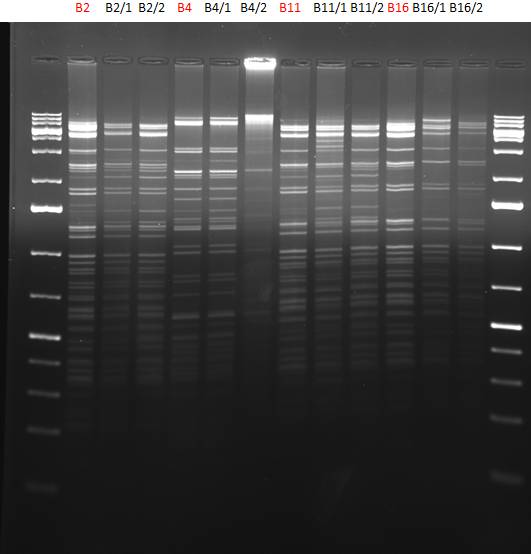


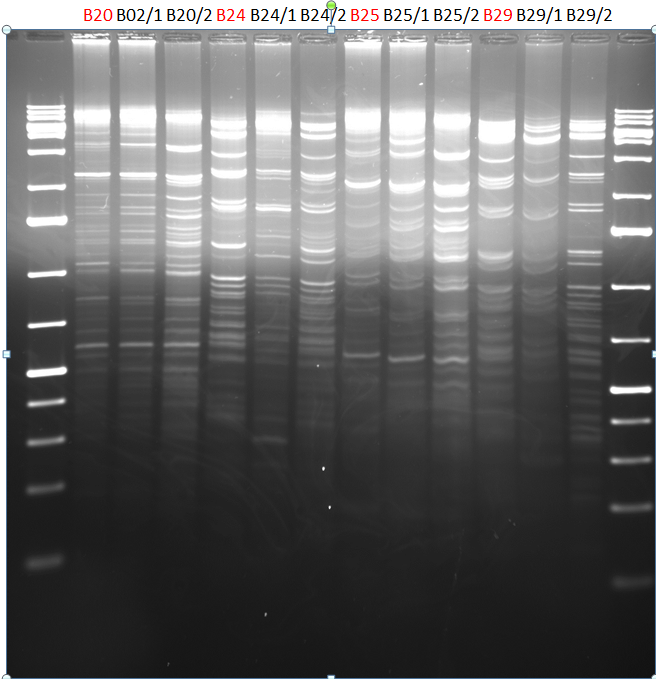


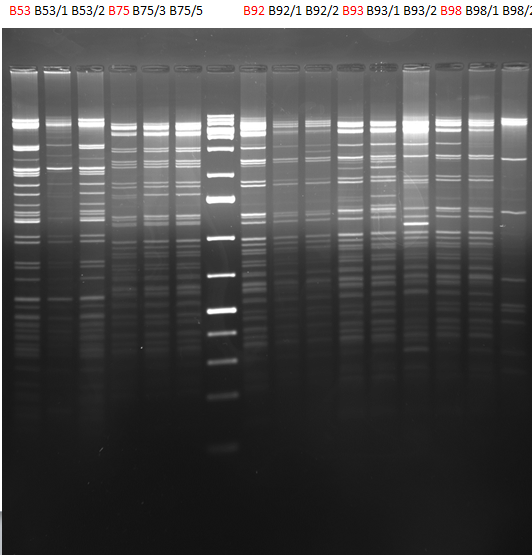

Supplement: Supplementary file 2 — Supplementary Material 2. [file 41598_2026_51924_MOESM2_ESM.docx]
